# Bacteria and sputum inflammatory cell counts; a COPD cohort analysis

**DOI:** 10.1186/s12931-020-01552-4

**Published:** 2020-11-01

**Authors:** Augusta S. Beech, Simon Lea, Umme Kolsum, Zhang Wang, Bruce E. Miller, Gavin C. Donaldson, Jadwiga A. Wedzicha, Christopher E. Brightling, Dave Singh

**Affiliations:** 1 Division of Infection, Immunity and Respiratory Medicine, School of Biological Sciences, Faculty of Biology, Medicine and Health, Manchester Academic Health Science Centre, The University of Manchester, Manchester, UK; 2 Medicines Evaluation Unit, University of Manchester, Manchester University NHS Foundation Trust, Southmoor Road, Manchester, M23 9QZ UK; 3 Institute of Ecological Sciences, School of Life Sciences, South China Normal University, Guangzhou, China; 4 Medical Innovation, Value Evidence and Outcomes, GSK R&D, Collegeville, PA USA; 5 National Heart and Lung Institute, Imperial College London, London, UK; 6 Institute for Lung Health, University of Leicester, Leicester, UK

**Keywords:** Microbiome, COPD, Haemophilus influenzae, Sputum, Eosinophil

## Abstract

**Background:**

There is evidence that bacterial colonisation in chronic obstructive pulmonary disease (COPD) is associated with increased neutrophilic airway inflammation. This study tested the hypothesis that different bacterial phyla and species cause different inflammatory profiles in COPD patients.

**Methods:**

Sputum was analysed by quantitative polymerase chain reaction (qPCR) to quantify bacterial load and 16S rRNA gene sequencing to identify taxonomic composition. Sputum differential cell counts (DCC) and blood DCC were obtained at baseline and 6 months. Patients were categorised into five groups based on bacterial load defined by genome copies/ml of ≥ 1 × 10^4^, no colonisation and colonisation by *Haemophilus influenzae* (*H. influenzae*), *Moraxella catarrhalis* (*M. catarrhalis*), *Streptococcus pneumoniae* (*S. pneumoniae*), or > 1 potentially pathogenic microorganism (PPM).

**Results:**

We observed an increase in sputum neutrophil (%), blood neutrophil (%) and neutrophil–lymphocyte ratio (NLR) in patients colonised with *H. influenzae* (82.6, 67.1, and 3.29 respectively) compared to those without PPM colonisation at baseline (69.5, 63.51 and 2.56 respectively) (p < 0.05 for all analyses), with similar findings at 6 months. The bacterial load of *H. influenzae* and *Haemophilus* determined by qPCR and 16s rRNA gene sequencing respectively, and sputum neutrophil % were positively correlated between baseline and 6 months visits (p < 0.0001, 0.0150 and 0.0002 with r = 0.53, 0.33 and 0.44 respectively).

**Conclusions:**

These results demonstrate a subgroup of COPD patients with persistent *H. influenzae* colonisation that is associated with increased airway and systemic neutrophilic airway inflammation, and less eosinophilic airway inflammation.

## Background

Chronic obstructive pulmonary disease (COPD) is a heterogeneous disease characterised by airflow obstruction and persistent airway inflammation [1]. COPD patients show increased susceptibility to bacterial infection, through mechanisms such as decreased bacterial phagocytosis [2]. Chronic bacterial airway colonisation may occur with potentially pathogenic microorganisms (PPMs) including *Haemophilus influenzae (H. influenzae), Moraxella catarrhalis (M. catarrhalis)* and *Streptococcus pneumoniae (S. pneumoniae)* [3–5].

The presence of bacterial infection in COPD patients during the stable state (i.e. not during exacerbations) is associated with increased airway neutrophil numbers [6, 7]*.* Furthermore*,* it appears that different bacterial phyla are associated with different profiles of airway inflammation in COPD patients; raised blood and sputum eosinophils are associated with increased presence of the Bacteroidetes phylum [8], while low sputum eosinophil levels have been associated with increased *H. influenzae* presence [9]. Additionally, a study of microbiome, transcriptome and proteome profiling showed that *H. influenzae* presence in the stable state was associated with a unique profile of inflammation, including increased sputum neutrophil counts [10, 11].

The analyses of randomised clinical trials have demonstrated that greater inhaled corticosteroid (ICS) benefits are observed at higher blood eosinophil counts [12]. Consequently, the Global initiative for the management of chronic Obstructive Lung Disease (GOLD) report recommends the use of blood eosinophil counts to guide ICS treatment in COPD patients with a history of exacerbations [1]. Higher blood eosinophil counts in COPD patients are associated with increased eosinophil numbers in bronchial tissue, broncho-alveolar lavage and sputum, increased levels of T2 cytokines and greater basement membrane thickening [13]. The mechanistic reasons for increased ICS effects at higher blood eosinophil counts may be related to an inflammatory profile associated with increased levels of T2 cytokines, but an association with a different microbiome profile may also be important [10].

Using the COPDMAP cohort, we have further studied the relationship between sputum cell counts and bacterial species in the stable state. We evaluated the stability of the relationship between the microbiome and airway inflammation using repeated samples at 6 months, primarily focusing on bacterial load measured by quantitative polymerase chain reaction (qPCR) and sputum cell counts. Associations between these parameters and both 16S rRNA-gene based microbiome analysis and blood leucocyte counts are also reported. A focus of the analysis of repeated samples was to investigate the persistence of bacterial colonisation over 6 months, and its associations with sputum cell counts and clinical characteristics.

## Methods

### Subjects

COPD patients aged ≥ 40 years were recruited at 3 sites (Manchester, Leicester and London) into the COPDMAP prospective observational cohort study. Patients had a physician diagnosis of COPD, post-bronchodilator forced expiratory volume in 1 s (FEV_1_)/forced vital capacity (FVC) ratio < 0.7, ≥ 10 pack year smoking history and no previous asthma diagnosis. The patients included in this analysis were those who provided a sputum sample for bacterial quantification at ≥ 1 visit over 12 months. All patients provided written informed consent using protocols approved by the local Ethics Committees (11/L0/1630; 10/H/1003/108; 07/H0406/157).

### Study design

Patient visits were during stable state at baseline and at 6 months (stable defined as no symptom-defined exacerbation in the 4 weeks preceding sampling). Patients who received maintenance oral corticosteroid or antibiotic therapy were excluded from analysis. Symptoms were assessed using the COPD assessment test (CAT) [14]. Health related quality of life was assessed using the St George’s respiratory Questionnaire (SGRQ-C) [15]. Lung function measurements were performed according to guidelines [16]. Spontaneous or induced sputum was obtained as previously described [17]. Blood samples were sent to local hospital laboratories for analysis of differential cell counts (DCC). A total of 236 patients were included in this analysis, the number of patients included in different sub-analyses was dependent on the availability of specific data relevant to that sub-analyses; 236 patients produced a sputum sample for bacterial analysis using qPCR at baseline; 226 had qPCR bacterial quantification and blood DCC; 145 had qPCR and sputum DCC. At 6 months, there were 100 patients with qPCR bacterial quantification and blood DCC, and 69 with qPCR and sputum DCC (Additional file 1, Fig. S1).

### Bacterial qPCR and 16S rRNA measurements

Sputum induction was attempted if an insufficient spontaneous sample was produced for the analysis, approximately 18% of cases in the current analysis. Spontaneous or induced sputum was processed for qPCR detection of absolute abundance for the following bacterial species; *H. influenzae, M. catarrhalis* and *S. pneumoniae* as previously described [4]. Where possible, sputum was also processed for differential cell counts [18]. Patients were termed ‘’persistently colonised’’ if qPCR results indicated two consecutive positive measurements over 6 months. Although qPCR detection of bacterial species was the primary analysis, sputum was also processed for 16S RNA-gene based microbiome analysis of bacterial taxonomy (see online supplement).

### Statistical analysis

Patients with bacterial load ≥ 1 × 10^4^ copies/ml defined by qPCR were categorised as positive for PPM colonisation [6], then split into five groups; No colonisation, *H. influenzae*, *S. pneumoniae*, *M. catarrhalis*, and two or more PPMs (> 1 PPMs). Similar analysis was performed using the threshold ≥ 1 × 10^6^ copies/ml. Statistical analysis for non-parametric data was performed using; Kruskal–Wallis test followed by post-test analysis with either Wilcoxon signed rank test or the Mann–Whitney U test (with correction for multiple analysis). Spearman’s correlation assessed associations between variables. *p* < 0.05 was considered statistically significant. Analyses were performed using GraphPad Prism version 7.00 (San Diego, USA). For 16S rRNA analysis, alpha diversity was assessed using Shannon index. Kruskal–Wallis test identified taxa with significantly different abundance across groups. Wilcoxon rank sum tests compared enrichment of other taxa present in *H. influenae* and *S. pneumoniae* groups. For beta diversity, composition dissimilarity was tested using the Bray–Curtis dissimilarity index. The false discovery rate (FDR) method adjusted *P *values for multiple testing and significantly differentially represented bacterial taxa were identified using edgeR [10].

## Results

### Study subjects

The baseline demography of the cohort (n = 236) is shown in Table 1; the mean post-bronchodilator FEV_1_ was 57.4% predicted, with no exacerbations in the previous year in 32.6% of patients, 1 exacerbation in 22.9% and ≥ 2 exacerbations in 44.5%. Mean CAT and total SGRQ scores were 18 and 47 respectively.

**Table 1:** Baseline demographics of patients enrolled onto this study

Characteristic	n = 236
Gender (% male)	74
Age	69.5 (8.3)
Smoking status (current %)	34
Pack years	47.5 [10–220]
BMI (kg/m^2^)	26.3 [17.3–47.0]
Exacerbations (1 year period)	1 [0–15]
Post FEV_1_ (L)	1.5 (0.6)
Post FEV_1_ (%)	57.4 (18.5)
CAT	18 (7.5)
SGRQ-C (total)	47.0 (18.2)
GOLD	2 [1–4]
1 (%)	12.0
2 (%)	52.1
3 (%)	29.1
4 (%)	6.8
LABA only (%)	2.5
LAMA only (%)	5.5
ICS only (%)	1.7
ICS + LABA (%)	13.1
ICS + LAMA (%)	1.3
LABA + LAMA (%)	2.1
Triple (%)	68.2
No inhaled medication (%)	5.5

Summaries are presented as percentages, mean (SD), median [range] as appropriate (n = 236^a^)

^a^At baseline the following data were not available for; n = 8 Saint Georges respiratory Questionnaire (SGRQ), n = 3 Chronic obstructive pulmonary disease assessment test (CAT), n = 2 forced expiratory volume in one second (FEV_1_)

### Baseline results

The cohort (n = 236) was divided into five groups using qPCR detection using a threshold ≥ 1 × 10^4^ genome copies/ml; No colonisation (*n* = 108), *H. influenzae* only (*n* = 41), *S. pneumoniae* only (*n* = 47), *M. catarrhalis* only (*n* = 4) and > 1 PPM (*n* = 36), referred to as NC, *H. influenzae, S. pneumoniae, M. catarrhalis* and > 1 PPM groups respectively. The *M. catarrhalis* group were excluded from baseline analysis due to a small sample size. The > 1PPM group consisted of patients with colonisation of two or three PPMs; 92% were colonised with *H. influenzae*, with 58% colonised with *H. influenzae* + *S. pneumoniae*, while 42% showed evidence of *M. catarrhalis* colonisation and 8.3% were colonised with all three bacterial species (for details see Additional file 1, Table S1). The total number of patients in the entire cohort with *M. catarrhalis* colonisation was 19 (8.1%). There was no difference in bacterial colonisation in frequent exacerbators (≥ 2 exacerbations in the previous year) compared to non-frequent exacerbators (Additional file 1, Table S2), or in patients using ICS compared to those not using ICS (data not shown).

#### Sputum cell counts

**Fig. 1:**
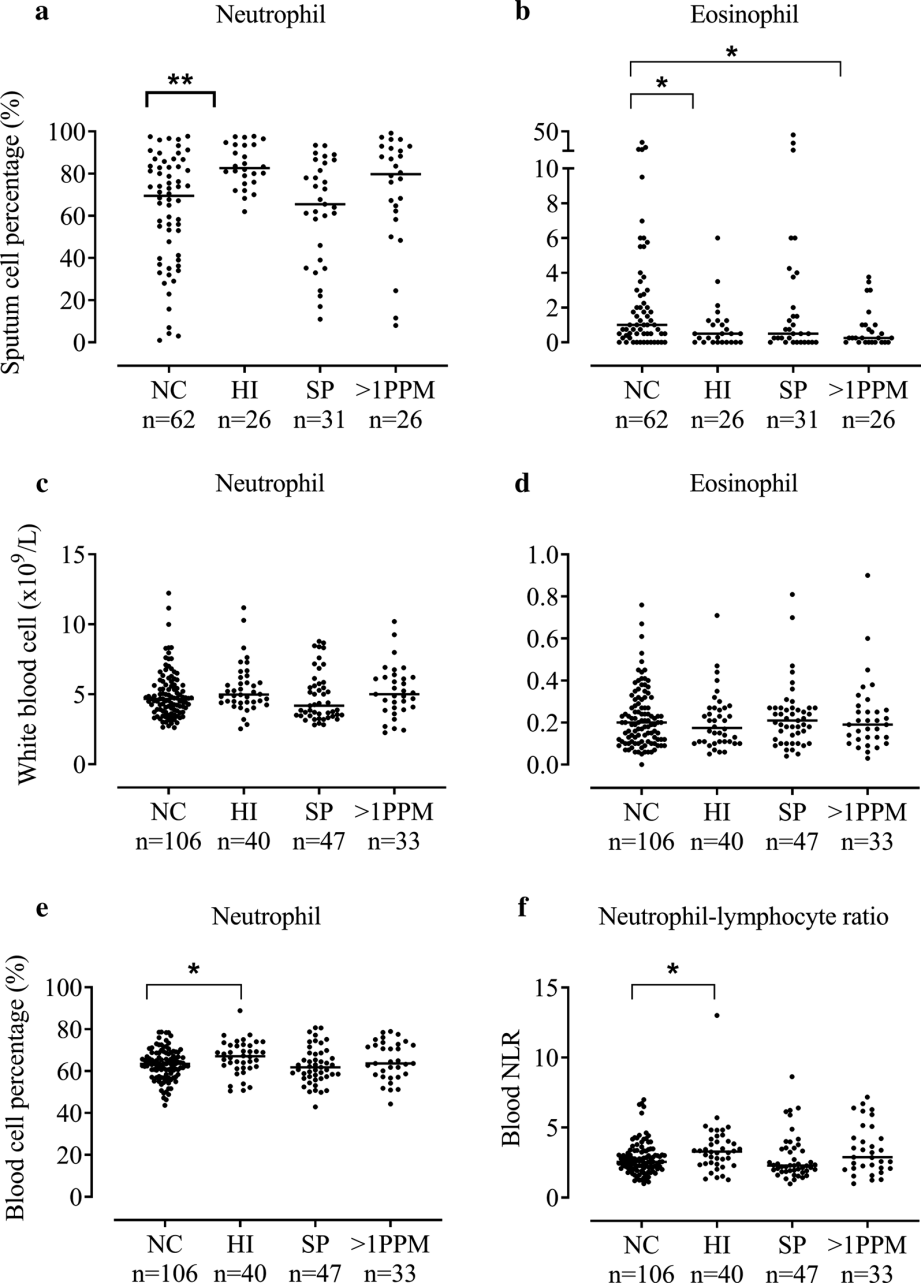
The comparison of sputum and blood eosinophil and neutrophil counts between patients colonised with different PPMs. Patients were categorised into four groups based on bacterial load defined by genome copies/ml of ≥ 1 × 10^4^; no colonisation (NC), colonised with *Haemophilus influenzae* (*HI*), *Streptococcus pneumoniae* (SP) or > 1 potentially pathogenic microorganism (PPM). Sputum neutrophil % (n = 145) (**a**), sputum eosinophil % (n = 145) (**b**), blood neutrophil counts (n = 226) (**c**), blood eosinophil counts (n = 226) (**d**), blood neutrophil percentages (n = 226) (**e**) and blood neutrophil–lymphocyte ratio (n = 226) (**f**) are shown for each group. Statistical analysis was performed using Kruskal–Wallis and Mann–Whitney U adjusted for multiple comparisons. Data represent individual patients with median. *, **Significant difference to no colonisation group (*p* < 0.05, < 0.01 respectively)

145 patients with qPCR data had sputum DCCs available; induced samples were more cellular based on total cell count (× 10^6^/g), with total neutrophil and macrophage counts (× 10^6^/g) also higher in induced samples in the subset with this information available (n = 112). However, in the total sample (n = 143) with available DCCs, the sputum cell percentages between induced and spontaneous samples were similar (Additional file 1, Table S3). There were no differences observed between qPCR and DCC results from induced and spontaneous samples (Additional file 1, Table S3). Sputum neutrophil % was higher in the *H. influenzae* versus NC group; medians 82.6 and 69.5% respectively (p = 0.0004, Fig. 1a). Neutrophil percentage was also higher in the > 1 PPM group (79.8%), but this difference was not statistically significant (p = 0.06). A similar pattern showing higher sputum neutrophil absolute cell counts in the *H. influenzae* versus NC group was evident; medians 8.59 × 10^6^/g versus 1.71 × 10^6^/g respectively (p = 0.0028, Additional file 1, Table S4).

The *H. influenzae* and > 1PPM groups had lower sputum eosinophil levels (medians 0.50% and 0.25% respectively) compared to the NC group (median 1.00%; p = 0.03 and 0.04 respectively, Fig. 1b). Using > 3% to define sputum eosinophilia, the *H. influenzae* and > 1PPM groups had fewer patients above this threshold (7.7% and 11.5% respectively) compared to NC (24.2%), although this difference was not significant (p = 0.17; Additional file 1, Table S4). No significant differences in absolute sputum eosinophil counts were observed between groups (Additional file 1, Table S4). The clinical characteristics of the groups showed some numerical differences; FEV_1_ predicted was lower in the *H. influenzae* and *S. pneumoniae* groups, while SGRQ was also higher in these groups (statistical analysis shown in Additional file 1, Table S5). There were no differences in ICS use between groups.

#### Blood cell counts

In 226 patients with blood samples, there were no significant differences between groups for absolute blood neutrophil or eosinophil cell counts (Fig. 1c, d, Additional file 1, Table S6) or eosinophil % (not shown). Blood neutrophil % and neutrophil–lymphocyte ratio were significantly higher in the *H. influenzae* versus NC group (medians: neutrophils 67.1% and 63.51% respectively, p = 0.0268; neutrophil–lymphocyte ratio: 3.32 and 2.56 respectively, p = 0.0086, Fig. 1e, f). There were no significant differences in blood lymphocyte counts between groups (Additional file 1, Fig. S2).

#### 16S rRNA microbiome analysis

**Fig. 2:**
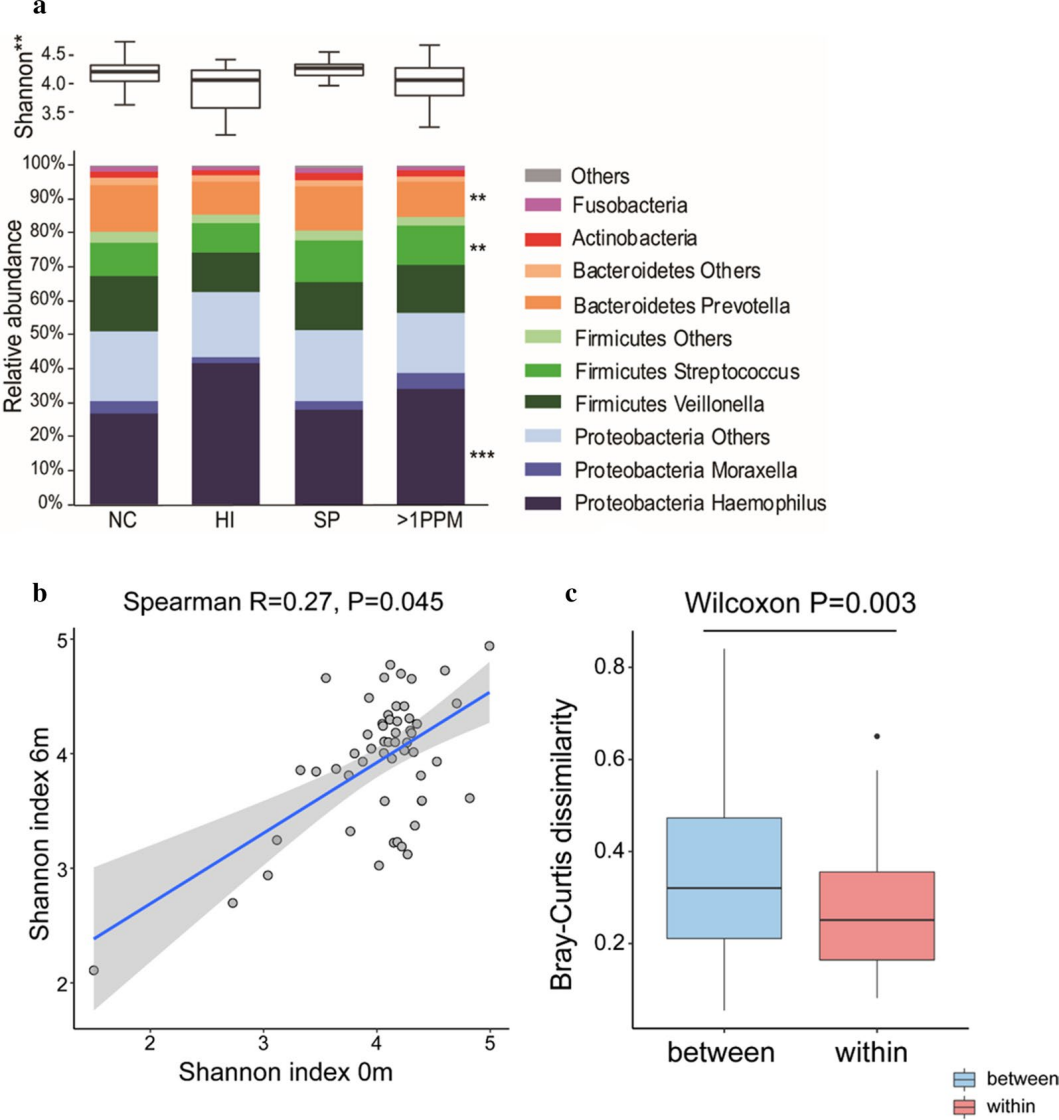
Sputum microbiome profiles in COPD patients at baseline. A proportion of patients provided sufficient sample for 16S rRNA sequence analysis. Shannon Diversity and relative abundance of major bacterial taxa and genera for PPM groups defined by genome copies per mL of ≥ 1 × 10^4^; no colonisation (NC), *Haemophilus influenzae* (*HI*), *Streptococcus pneumonia* (SP) and > 1PPM n = 153 (**a**), Correlation analysis for shannon diversity of paired samples at baseline and 6 months (n = 54) (**b**), Composition dissimilarity analysis between paired samples at baseline and 6 months, and samples from different individuals (n = 54) (**c**). *, **, ***Significant difference to no colonisation group (FDR *p* < 0.05, < 0.01 and < 0.001 respectively)

Microbiome profiles for 153 patients defined using baseline qPCR results are presented in Fig. 2. An increase in relative abundance of *Haemophilus* (FDR p = 0.0004) and decrease in *Prevotella* (FDR p = 0.003) and *Streptococcus* (FDR p = 0.007) was observed in both *H. influenzae* and > 1 PPM groups compared the NC group (Fig. 2a). In contrast, the *S. pneumoniae* group was enriched with numerous other bacterial phyla including other Fusobacteria (*Fusobacterium*), Bacteroidetes (*Prevotella*), Proteobacteria (*Campylobacter* and *Neisseria*) and Actinobacteria (*Rothia* and *Actinomyces*) (FDR p < 0.05). There were significant differences in Shannon indexes between groups; alpha diversity decreased in both *H. influenzae* and > 1PPM groups compared to NC. When analysed in relation to sputum (n = 128) and blood cell counts (n = 153), Shannon diversity was negatively associated with sputum neutrophil % (p < 0.01, rho = − 0.24) and absolute counts (p = 0.05, rho = − 0.21) but not blood neutrophils (data not shown).

### 6 month results

#### Sputum cell counts

**Fig. 3:**
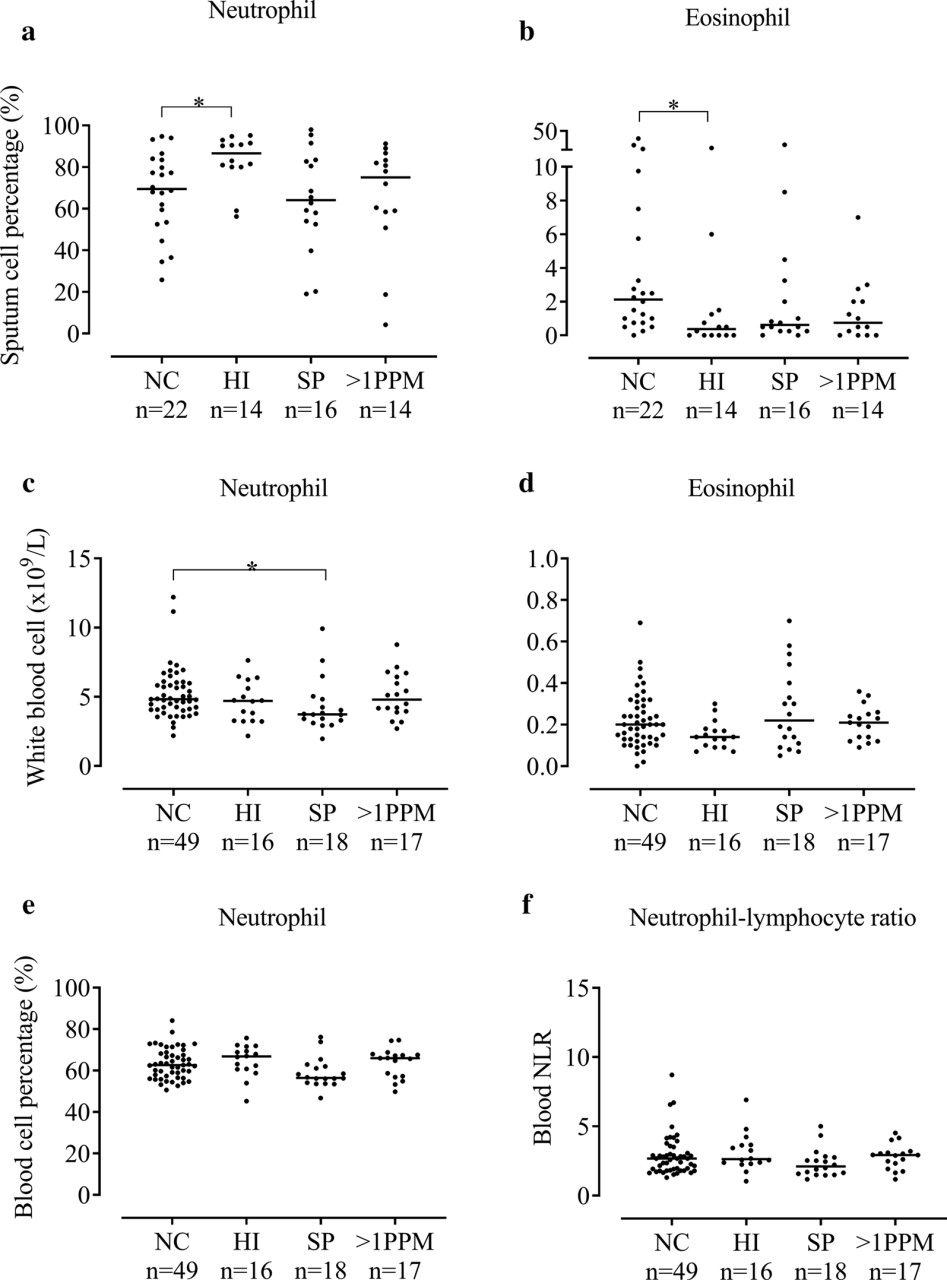
The comparison of sputum and blood eosinophil and neutrophil counts between patients colonised with different PPMs at 6 months post-baseline. Patients were categorised into four groups based on bacterial load defined by genome copies/ml of ≥ 1 × 10^4^; no colonisation (NC), colonised with *Haemophilus influenzae* (*HI*), *Streptococcus pneumoniae* (SP) or > 1 potentially pathogenic microorganism (PPM). Sputum neutrophil % (n = 66) (**a**), Sputum eosinophil % (n = 66) (**b**). Three patients were excluded due to colonisation with *Moraxella catarrhalis* at 6 months. Blood neutrophil count (n = 100) (**c**), Blood eosinophil count (n = 100) (**d**) Blood neutrophil % (n = 100) (**e**) and Blood neutrophil–lymphocyte ratio (n = 100) (**f**). Statistical analysis was performed using Kruskal–Wallis and Mann–Whitney U adjusted for multiple comparisons. Data represent individual patients with median. *Significant difference to no colonisation group (*p* < 0.05 respectively)

66 patients with qPCR data had sputum cell counts available. The *H. influenzae* group had significantly higher neutrophil percentages compared to the NC group (medians 86.6% and 69.5% respectively, p = 0.02, Fig. 3a), with a similar pattern for absolute neutrophil counts (12.89 and 1.45 × 10^6^/g respectively, p = 0.0048, Additional file 1, Table S7). The *H. influenzae* group had lower sputum eosinophil percentages compared to the NC group (0.38% and 2.13% respectively, p = 0.011, Fig. 3b), with no significant differences for absolute counts.

#### Blood cell counts

In 100 patients with blood samples, there were no consistent differences between groups for neutrophils, eosinophils or neutrophil–lymphocyte ratio (Fig. 3c–f, Additional file 1, Table S8).

#### Analysis using alternative qPCR threshold

An analysis using a threshold of ≥ 1 × 10^6^ copies/ml to define colonisation showed a similar pattern of results to ≥ 1 × 10^4^ copies/ml, at both baseline and 6 months; details are in the supplement (Additional file 1, Figs. S3, S4 and Tables S9, S10, S11, S12 and S13).

### Stability over 6 months

**Fig. 4:**
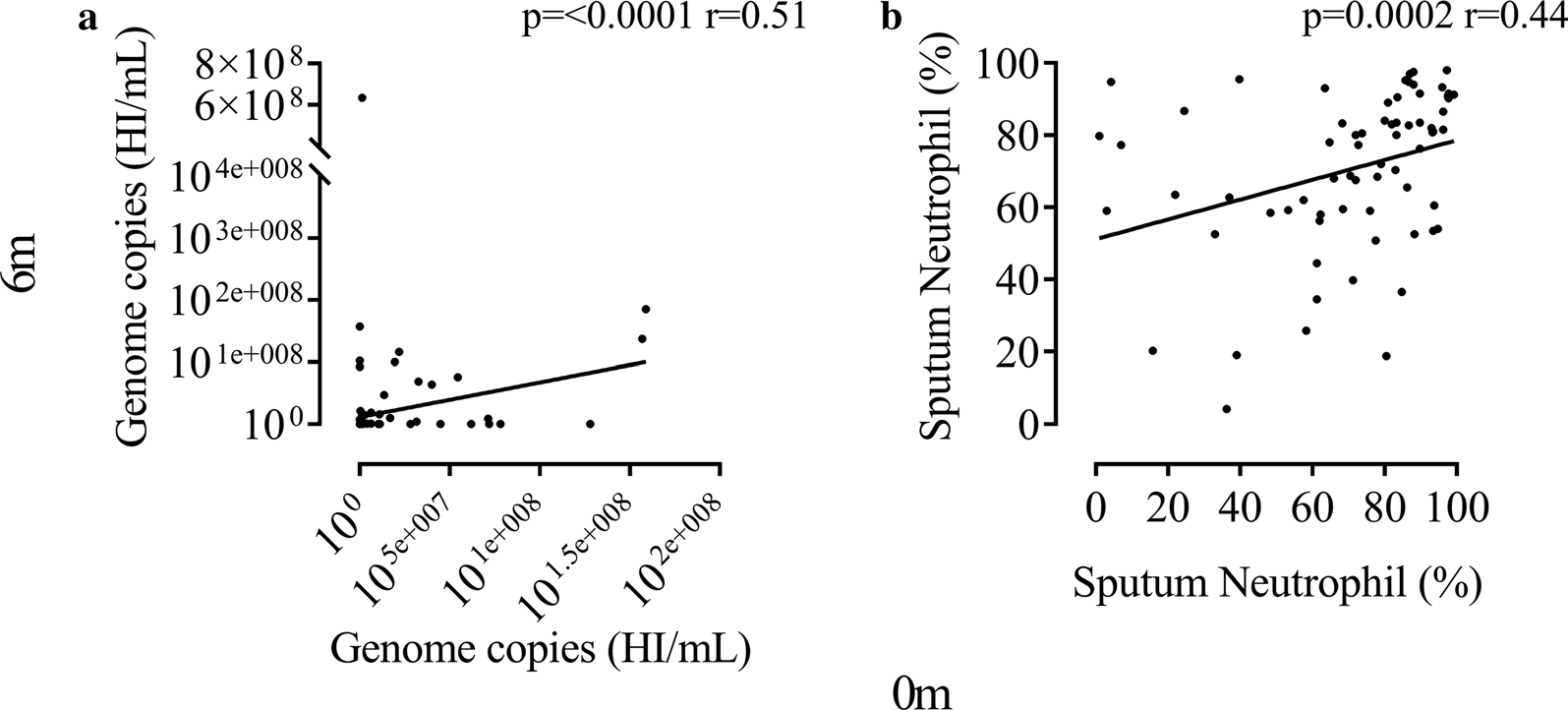
Stability of *Haemophilus influenzae* presence and airway neutrophil % over 6 months. Scatter correlation of *Haemophilus influenzae* (HI) genome copies/mL at baseline versus 6 months thereafter (n = 122) (**a**). Scatter correlation of sputum neutrophil percentage at baseline versus 6 months thereafter (n = 69) (**b**). Statistical analysis was performed using Spearmans Rank order correlation. Data represent individual patients with linear regression

The bacterial load of *H. influenzae* determined by qPCR at baseline and 6 months was positively correlated (n = 122; rho = 0.51, *p* < 0.0001, Fig. 4a). Associations at baseline and 6 months were also observed using *Haemophilus* abundance determined by 16S rRNA gene sequencing (n = 54; rho = 0.33, p = 0.015, Additional file 1, Fig. S6) and for sputum neutrophil % (n = 69; r = 0.44, p = 0.0002, Fig. 4b).

**Fig. 5:**
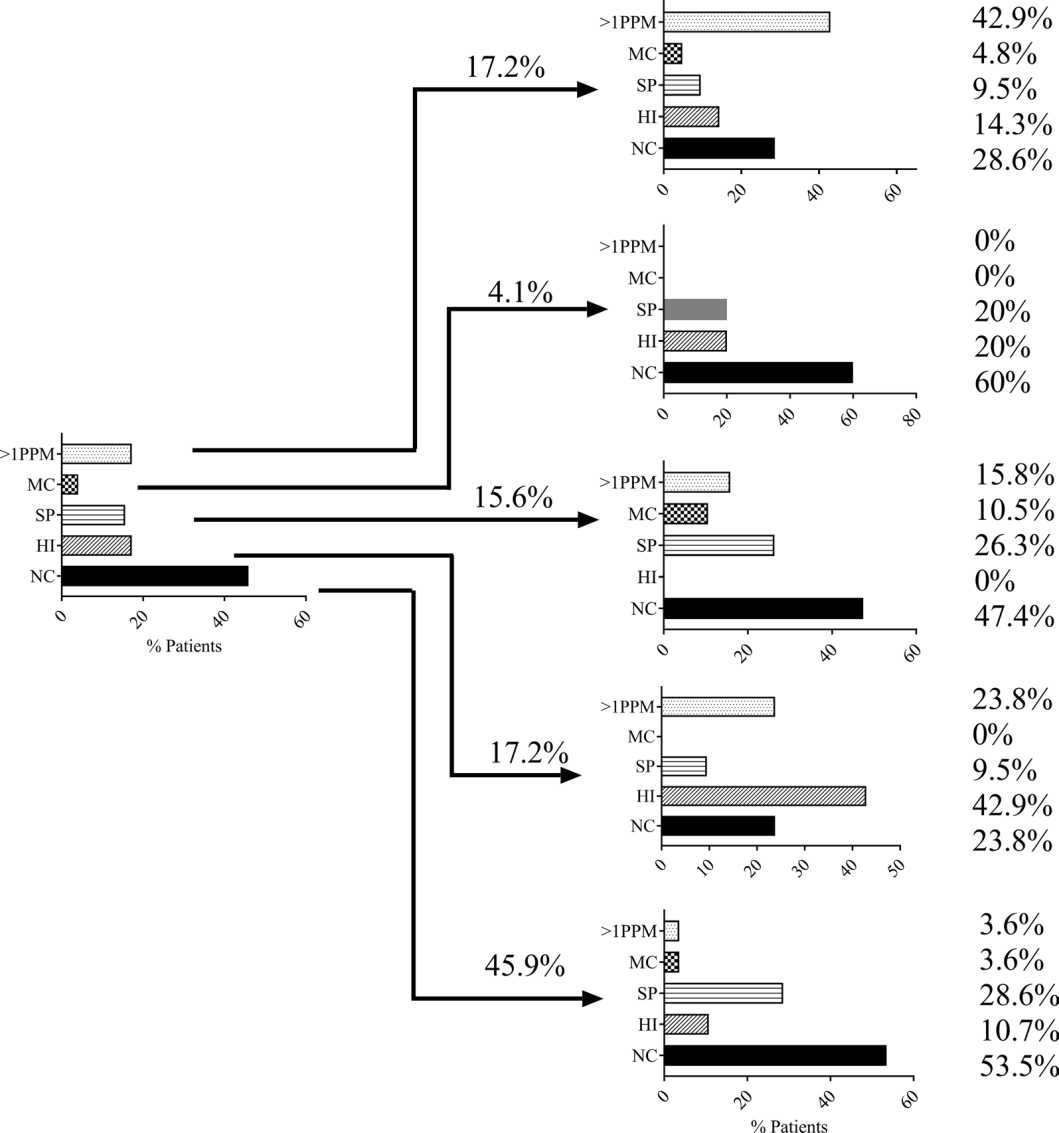
Changes in airway colonisation of respective potentially pathogenic microorganisms over 6 months. Patients were categorised into five groups based on bacterial load defined by genome copies/ml of ≥ 1 × 10^4^; No colonisation (NC) (45.9%), *Haemophilus influenzae* (HI) (17.2%), *Streptococcus pneumoniae* (SP) (15.6%), > 1 potentially pathogenic microorganism (PPM) (17.2%) or *Moraxella catarrhalis* (MC) (4.1%) (n = 122). Within groups, PPM classification at 6 months follow up is shown. 53.5, 42.9, 26.3, 42.9 and 0% of patients were recorded in the same group as baseline for no colonisation, HI, SP, > 1PPM and MC respectively

The changes in colonisation from baseline to 6 months (n = 122) are shown in Fig. 5. The most common findings at baseline were *H. influenzae* (*n* = 21, 17.2%), NC (*n* = 56, 45.9%) or > 1PPM (*n* = 21, 17.2%). For these groups, approximately 40–50% of patients remained in the same category at 6 months. Less patients in the *S. pneumoniae* and *M. catarrhalis* groups remained in the same category.

**Fig. 6:**
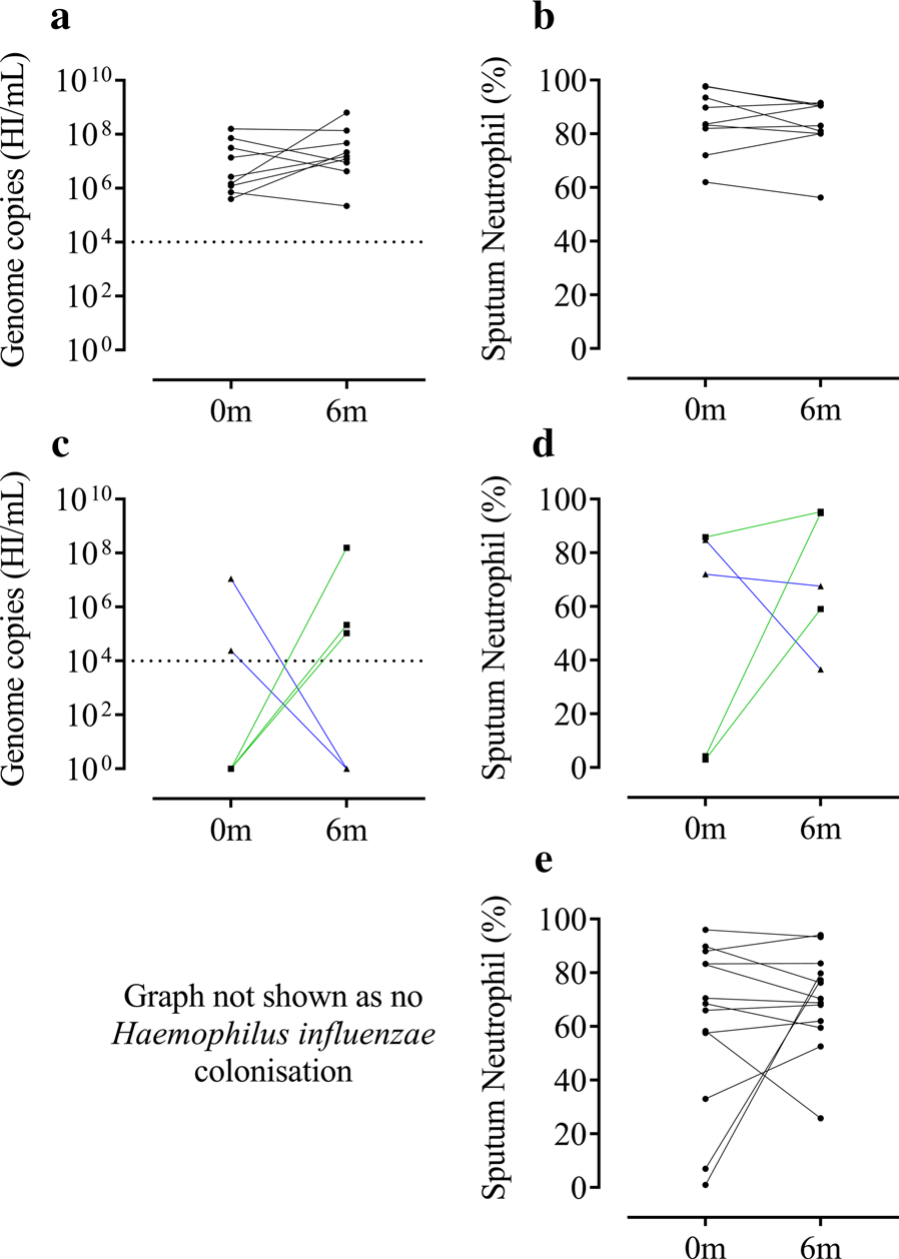
Levels of *Haemophilus influenzae* and sputum neutrophils at baseline and 6 months later. Change in *Haemophilus influenzae* (HI) level over 6 months for patients colonised with HI at both visits (n = 9) (**a**). Sputum neutrophil % over 6 months corresponding to patients in panel 6a (**b**). Change in HI level over 6 months for patients with development or loss of HI colonisation over 6 months (n = 5) (**c**). Sputum neutrophil % over 6 months corresponding to patients in panel **c** (n = 5) (**d**). Sputum neutrophil % over 6 months corresponding to patients with no colonisation at both visits (n = 13) (**e**). Green lines indicate gain and blue line indicates loss of HI colonisation over 6 months. Corresponding graph for panel 6e not shown as colonisation was below the lower level of detection. Dotted line indicates lower level of detection for bacterial qPCR of ≥ 1 × 10^4^ genome copies/mL (**a**, **c**). No formal statistical analyses were undertaken

Analysing patients with qPCR and sputum counts at both baseline and 6 months (n = 69), there were 9 with *H. influenzae* colonisation (Fig. 6a) and 30 with no colonisation at both visits (not shown). Patients colonised at both visits (termed “persistent colonisation”) had sputum neutrophil % that mostly remained above 60% at both visits, while non-colonised patients had a greater spread of neutrophil counts (Fig. 6b, e respectively). Patients with persistent *H. influenzae* presence had a significantly lower FEV_1_ compared to those without *H. influenzae* colonisation (46% versus 61.2% predicted respectively, p = 0.035; Additional file 1, Table S14 shows clinical characteristics including exacerbations rates which were not significantly different). Five patients were colonised with *H. influenzae* at 1 visit only (Fig. 6c); patients who developed new *H. influenzae* colonisation showed an increase in sputum neutrophil %, while a loss of *H. influenzae* colonisation was associated with a reduction in sputum neutrophil % (Fig. 6d). Patients persistently colonised with *H. influenzae* had higher sputum neutrophil and lower sputum eosinophil (%) (p = 0.011 and 0.016 respectively; Additional file 1, Table S15). A further 16 patients were persistently colonised with *H. influenzae* + ≥ 1 other PPM at both baseline and 6 months visits. We combined these 16 patients with the 9 patients colonised with *H. influenzae* only, and compared this group (n = 25) to non-colonised patients. Again, there was a significantly lower FEV_1_% predicted in *H. influenzae* colonised patients compared to those without colonisation (p = 0.01), but no difference in exacerbation rates (Additional file 1, Table S16). Sputum neutrophil % was significantly higher in *H. influenzae* persistently colonised patients and sputum eosinophil % was lower (p < 0.01 and 0.03 respectively. Additional file 1, Table S17). Using eosinophil % thresholds, a significantly lower proportion of eosinophilic patients were observed in the *H. influenzae* persistently colonised group (p = 0.01 and < 0.01 using 2 and 3% sputum eosinophil thresholds respectively).

The 6 month data for 16S rRNA analysis showed a similar pattern to the baseline data but with a smaller sample size (n = 73) (Additional file 1, Figs. S5 and S6). The Shannon diversity for paired samples at baseline and 6 m showed a positive correlation (n = 54) (Spearman R = 0.27, p = 0.045, Fig. 2b). We tested the composition dissimilarity between samples from the same individual at baseline and 6 months, and samples from different individuals. The samples from the same patients had significantly smaller Bray–Curtis dissimilarity indices than those from different individuals (n = 54) (Wilcoxon p = 0.003, Fig. 2c), suggesting relative temporal stability of the airway microbiome in COPD.

## Discussion

This analysis of the COPDMAP cohort demonstrated a subgroup of COPD patients with *H. influenzae* colonisation in the stable state, associated with increased neutrophil and decreased eosinophil numbers in sputum. Although colonisation status and microbiome changed over time in some patients, we observed that approximately 40% of patients within the *H. influenzae* group at baseline had persistent *H. influenzae* colonisation at 6 months, with a similar profile of airway inflammation. *H. influenzae* colonisation was also associated with increased blood neutrophils numbers. In contrast, there was no association between neutrophilic inflammation and *S. pneumoniae* colonisation. These results reveal a distinct COPD subgroup with *H. influenzae* colonisation that persists over time and is associated with increased neutrophilic inflammation in both the lungs and systemic circulation.

The 16S rRNA microbiome results supported the qPCR analysis, showing increased *Haemophilus* abundance in the *H. influenzae* group defined by qPCR at baseline, with no other genus enriched in this group. In contrast, a variety of other bacterial genus (not *Haemophilus*) were enriched in the *S. pneumoniae* group. Additionally, most of the > 1PPM group (92%) were colonised with *H. influenzae,* and there was a trend towards increased sputum neutrophil counts in this group. Overall, these qPCR and 16S rRNA results indicate that *Haemophilus* colonisation may occur with or without the presence of other colonising bacteria, but that the presence of *H. influenzae* is the key component causing the association with neutrophil counts.

*H. influenzae* and > 1PPM groups displayed the greatest stability over 6 months compared to *S. pneumoniae* and *M. catarrhalis*. It was not possible to determine if the changes in inflammatory cell counts were solely attributed to the presence of *H. influenzae* or rather the persistence of airway colonisation in these patients, although there is evidence to support the former [10]. Significant associations were demonstrated using both qPCR and 16s rRNA gene sequencing for *Haemophilus* measurements at baseline and 6 months. Sputum neutrophils showed a similar pattern. Changes in airway neutrophils on an individual basis over time showed remarkable concordance with that of *H. influenzae* colonisation in the subgroup (n = 69) with sputum cell counts and microbiology data at both baseline and 6 months. The presence of *H. influenzae* appeared to influence the sputum neutrophil percentage, with persistently higher neutrophils being observed in those with *H. influenzae* present at both visits, and concordant changes in neutrophils observed when *H. influenzae* presence changed (Fig. 6). We also observed that persistent *H. influenzae* colonisation was associated with a lower FEV_1_ predicted, and a numerically higher exacerbation rate (although this difference was not statistically significant, possibly due to the small sample size, n = 9). The clinical phenotype relating to persistent colonisation with *H. influenzae* is likely to be of important clinical relevance and warrants further investigation in larger longitudinal cohort analysis.

Previous studies have shown associations between total airway bacterial load and neutrophilic inflammation using sputum [6, 19] and broncho-alveolar lavage [20] samples. *H. influenzae* presence in stable state sputum samples has been associated with increased sputum neutrophil counts [21, 22] and other pro-inflammatory biomarkers [19]. The present study confirms these observations regarding *H. influenzae,* and extends our knowledge by showing that *H. influenzae* persists in the stable state in a COPD subgroup, and is associated with persistent sputum neutrophilic inflammation and a skewing away from eosinophilic inflammation. Furthermore, we show an association between *H. influenzae* and systemic neutrophilic inflammation. Increased systemic inflammation is associated with co-morbidities including cachexia and cardiovascular disease, highlighting the potential clinical importance of our findings [23, 24].

Some COPD studies have shown that greater neutrophilic airway inflammation in the stable state is associated with poor clinical outcomes [25], including FEV_1_ decline [26], while other studies have reported little convincing evidence of any relationship [27]. Perhaps the variation between studies is due to differences between populations in the extent of bacterial colonisation with *H. influenzae* which is known to increase exacerbation susceptibility, particularly when exposure to human rhinovirus occurs [28].

Clinical trials have shown that lower sputum eosinophil numbers are associated with a reduced response to corticosteroid treatment [29]. The clinical use of blood eosinophils to direct ICS treatment is based on the association between blood and lung eosinophil numbers [12, 13]. Our findings implicate *H. influenzae* presence as a determinant of ICS response, as this bacterium skews the airway inflammation profile away from eosinophilic inflammation which is more ICS responsive. Furthermore, it has recently been reported that lower blood eosinophil counts are associated with increased chronic bacterial infection [30]. *H. influenzae* causes secretion of CXCL8 (a neutrophil chemokine) from alveolar macrophages in a corticosteroid insensitive manner in response to exposure [31], potentially explaining the observed skewing of neutrophil relative to eosinophil numbers.

*H. influenzae* can persist in the lung through various evasive pathogenic features, for example by formation of biofilms and resistance to neutrophil extracellular traps [32, 33]. The strains of *H. influenzae* present in the lungs of COPD patients may change over time [34]; our analysis does not define whether *H. influenzae* persistence at both baseline and 6 months are the same or different strains. Regardless, increases in neutrophilic airway inflammation associated with *H. influenzae* colonisation at baseline and 6 month visits were similar.

It has been reported that increased blood neutrophil numbers are associated with increased pneumonia risk in COPD patients [35]. These reports are compatible with the links between infection, microbiome dysbiosis and systemic inflammation reported here. Although we report only a relative increase in systemic neutrophil levels, the neutrophil–lymphocyte ratio (NLR) has received growing interest as a marker of systemic inflammation [36], and as a marker of severity for sepsis, pneumonia and cardiovascular diseases [36, 37]. We found the NLR to be significantly higher in those colonised with *H. influenzae*, further supporting a role for the species in driving systemic inflammation and pneumonia risk.

*M. catarrhalis* has been previously described as an exacerbation-related opportunistic pathogen [5, 10]. We report a lower stable state prevalence of *M. catarrhalis* (8.1%) than previous studies reporting 16% and 11% prevalence [38, 39]. These previous studies reported greater exacerbation frequencies prior to sampling (mean/year 3.6 and 3.5 [38, 39] compared with 1.89 in our analysis), possibly explaining the differences.

A limitation of this study is the reduced sample size during repeat sampling due to patient drop outs, exacerbations causing delayed stable visits and failure to produce a sputum sample at every visit. A large proportion of sputum samples analysed were spontaneously produced (83% and 88% at baseline and 6 months respectively). Previous studies have reported no differences in sputum cell percentages between induced and spontaneous samples [6, 17, 40], with our results agreeing with these findings. We observed a higher total cell count, and total neutrophil and macrophage counts in induced samples. Some caution should be applied not to over-interpret these findings, as the number of induced samples was much smaller, and no difference in total cell counts has been previously reported when paired samples from the same subjects were analysed [40]. Furthermore, this finding for total cell counts was not associated with any differences in bacteriology data. Nevertheless, our findings suggest the possibility that sputum induction results in more cellular samples compared to spontaneously obtained sputum, and this may have been a source of variation in this study [41]. Also, a large proportion of patients reported current use of ICS. It has been reported that higher ICS doses are significantly associated with greater total airway bacterial load [4, 7], but we did not see a difference in ICS use between groups, perhaps due to the high overall ICS use. Some patients were current smokers (34%); a previous analysis of the COPDMAP data reports no difference in microbiome between smoking and non-smoking individuals [42]. We were unable to determine if any changes in colonisation status were affected by antibiotics or prior vaccination. Finally, our analyses did not extend to investigation of the molecular mechanisms pertaining to the relationship between *H. influenzae* colonisation and neutrophilic inflammation.

## Conclusion

In conclusion, we observed a subgroup of COPD patients with persistent *H. influenzae* colonisation; this was associated with increased airway and systemic neutrophilic airway inflammation, and less eosinophilic airway inflammation. Although these results do not confirm causality, they highlight the possibility that *H. influenzae* may have a unique influence on inflammatory cells of the airway and circulation in COPD patients.

## Supplementary information

**Additional file 1: Figure S1.** Flow chart of subject selection process. **Figure S2.** The comparison of Blood Lymphocyte counts between patients colonised with different PPMs at 6 months post-baseline. **Figure S3.** The comparison of sputum and blood eosinophil and neutrophil counts between patients colonised with different PPMs. **Figure S4.** The comparison of sputum and blood eosinophil and neutrophil counts between patients colonised with different PPMs at 6 months post-baseline. **Figure S5.** Sputum microbiome profiles in COPD patients at 6 months. **Figure S6.** Stability of *Haemophilus* presence over 6 months as defined by 16s rRNA Sequencing. **Table S1.** Composition of > 1PPM group at baseline. **Table S2.** Sputum inflammatory cell counts and qPCR for patients with frequent or infrequent exacerbations in the 12 months prior to baseline. **Table S3.** Baseline sputum inflammatory cell counts for induced versus spontaneous sputum samples. **Table S4.** Baseline sputum inflammatory cell counts for different PPM groups. **Table S5.** Baseline Demographics of patients enrolled onto this study for different PPM groups. **Table S6.** Baseline blood inflammatory cell counts for different PPM groups. **Table S7.** Sputum inflammatory cell counts for different PPM groups at 6 months. **Table S8.** Blood inflammatory cell counts for different PPM groups at 6 months. **Table S9.** Baseline sputum inflammatory cell counts for different PPM groups, × 10^6^ copies/ml to detect PPMs. **Table S10.** Baseline blood inflammatory cell counts for different PPM groups, × 10^6^ copies/ml to detect PPMs. **Table S11.** Sputum inflammatory cell counts for different PPM groups at 6 months, × 10^6^ copies/ml to detect PPMs. **Table S12.** Blood inflammatory cell counts for different PPM groups at 6 months, × 10^6^ copies/ml to detect PPMs. **Table S13.** Baseline demographics, sputum and blood inflammatory cell counts for different PPM groups, × 10^6^ copies/ml to detect PPMs. **Table S14.** Baseline demographics for different PPM groups. **Table S15.** Sputum and blood inflammatory cell counts for different PPM groups. **Table S16.** Baseline demographics for different PPM groups, including those with *H. influenzae* + ≥ 1 PPM. **Table S17.** Baseline sputum inflammatory cell counts for different PPM groups, including those with *H.influenzae* + ≥ 1 PPM. **Table S18.** Occurrence and average relative abundance of contaminate genera detected in sequenced negative ‘blank’ controls by Salter et al. [5] in the COPDMAP dataset.

## Data Availability

The datasets generated and/or analysed during the current study are not publicly available.

## Abbreviations

COPD: Chronic obstructive pulmonary disease
CAT: COPD assessment test
DCC: Differential cell count
FEV_1_: Forced expiratory volume in 1 s
GOLD: Global initiative for the management of chronic obstructive pulmonary disease
*H. influenzae*: *Haemophilus influenzae*
ICS: Inhaled corticosteroids
*M. catarrhalis*: *Moraxella catarrhalis*
NLR: Neutrophil–lymphocyte ratio
NC: No colonisation
PPM: Potentially pathogenic microorganism
qPCR: Quantitative polymerase chain reaction
*S. pneumoniae*: *Streptococcus pneumoniae*
SGRQ: St George’s respiratory questionnaire
