# Supplementary material for: Bacteria and sputum inflammatory cell counts; a COPD cohort analysis
Source: Respir Res. 2020 Nov 1;21:289. doi: 10.1186/s12931-020-01552-4 (PMC7603729; doi:10.1186/s12931-020-01552-4)
Supplement: Supplementary file 1 — Additional file 1: Figure S1. Flow chart of subject selection process. Figure S2. The comparison of Blood Lymphocyte counts between patients colonised with different PPMs at 6 months post-baseline. Figure S3. The comparison of sputum and blood eosinophil and neutrophil counts between patients colonised with different PPMs. Figure S4. The comparison of sputum and blood eosinophil and neutrophil counts between patients colonised with different PPMs at 6 months post-baseline. Figure S5. Sputum microbiome profiles in COPD patients at 6 months. Figure S6. Stability of Haemophilus presence over 6 months as defined by 16s rRNA Sequencing. Table S1. Composition of > 1PPM group at baseline. Table S2. Sputum inflammatory cell counts and qPCR for patients with frequent or infrequent exacerbations in the 12 months prior to baseline. Table S3. Baseline sputum inflammatory cell counts for induced versus spontaneous sputum samples. Table S4. Baseline sputum inflammatory cell counts for different PPM groups. Table S5. Baseline Demographics of patients enrolled onto this study for different PPM groups. Table S6. Baseline blood inflammatory cell counts for different PPM groups. Table S7. Sputum inflammatory cell counts for different PPM groups at 6 months. Table S8. Blood inflammatory cell counts for different PPM groups at 6 months. Table S9. Baseline sputum inflammatory cell counts for different PPM groups, × 106 copies/ml to detect PPMs. Table S10. Baseline blood inflammatory cell counts for different PPM groups, × 106 copies/ml to detect PPMs. Table S11. Sputum inflammatory cell counts for different PPM groups at 6 months, × 106 copies/ml to detect PPMs. Table S12. Blood inflammatory cell counts for different PPM groups at 6 months, × 106 copies/ml to detect PPMs. Table S13. Baseline demographics, sputum and blood inflammatory cell counts for different PPM groups, × 106 copies/ml to detect PPMs. Table S14. Baseline demographics for different PPM groups. Table S15. Sputum and bloo [file 12931_2020_1552_MOESM1_ESM.docx]

**Additional Data Supplement**

**Bacteria and sputum inflammatory cell counts; a COPD cohort analysis**

Augusta S Beech, Simon Lea, Umme Kolsum, Zhang Wang, Gavin C Donaldson, Jadwiga A Wedzicha, Christopher E Brightling, Bruce E Miller, Dave Singh

**Methods**

**Microbiome processing and analyses**

The COPDMAP 16S rRNA gene datasets were deposited at the National Centre for Biotechnology Information Sequence Read Archive (SRP102480) as previously described ([1](#_ENREF_1)). The 16S rRNA gene datasets were processed using a standardized pipeline in QIIME 2.0 ([2](#_ENREF_2)). The sequencing reads were quality filtered using cut adapt ([3](#_ENREF_3)) (q=20, m=20). The quality-filtered reads were demultiplexed and denoised to generate high quality amplicon sequence variants (ASVs) using the Divisive Amplicon Denoising Algorithm 2 (DADA2) with default parameters ([4](#_ENREF_4)). In contrast to traditional OTU clustering-based methods, DADA2 used a model-based, clustering-free approach to correct amplicon errors and group unique sequences into ASVs. Hence it is thought to identify taxonomic variations at a finer-scale. For taxonomy classification of ASVs, a custom Naïve Bayes classifier was trained on the Greengenes 13_8 99% OTUs for each specific dataset according to its primers used and the length of the amplicon and applied to assign taxonomy for the ASVs in the dataset. The resulted OTU table was rarefied at the depth of 48191 reads for all samples. Singletons, mitochondria and chloroplast ASVs were filtered.

Negative controls for extraction (no sputum material) and PCR amplification (no template, Qiagen Elution Buffer only) were included in the experiment and sequenced, as described previously ([4](#_ENREF_4)). We performed further analyses to ensure that potential contamination risks were minimized. We compared our results against the 92 contaminant genera detected in sequenced negative ‘blank’ controls by Salter et al ([5](#_ENREF_5)). We failed to detect 42 out of the 92 contaminant genera in our dataset (Table S18). Of the remaining genera that were found in our data, none had an average relative abundance greater than 0.002, or had a relative abundance greater than 0.1 in any particular sample, except for Pseudomonas and Streptococcus which are known lung pathogens (Table S18).

**Results**

*Sputum cell counts*

Where possible, both absolute and differential cell counts were analysed. In some cases individual cell count data per gram was missing due to administrative errors; n=31 out of 145 and n=6 out of 69 were missing cell count data per gram at baseline and 6 months respectively.

***Analysis using alternative qPCR threshold***

***Baseline results***

The sputum microbiology showed that the cohort (n=236) were split into 5 groups defined by the presence of PPM (≥1x10^6^ genome copies/ml); No colonisation 58.9% (n=139), *H.influenzae* only 10.6% (n=25), *S.pneumoniae* only 17.4% (n=41), *M.catarrhalis* only 2.1% (n=5) and >1 PPM 11.0% (n=26). Patients colonised with *M.catarrhalis* only were excluded from this part of the analysis due to a small sample size. The >1PPM group consisted of patients with colonisation of two or three PPMs; 88.5% were colonised with *H.influenzae*, with 53.9% colonised with *H.influenzae* + *S.pneumoniae*, while 42.3% showed evidence of *M.catarrhalis* colonisation and 3.9% were colonised with all three bacterial species. The total number of patients in the entire cohort with *M.catarrhalis* colonisation was n=16 (6.8%).

*Sputum cell counts*

145 patients with qPCR data had sputum cell counts available. Sputum neutrophil percentage was higher in patients colonised with *H.influenzae* compared to those without colonisation; median neutrophil % 83.4 and 72.0 respectively (p<0.01, Figure S3A). Neutrophil percentage was also higher in the >1PPM group (82.1%), but this difference was not statistically significant (p=0.12). A similar pattern showing higher median sputum neutrophil absolute cell counts in the *H.influenzae* group was evident; 8.26 x10^6^/g versus 2.03 x10^6^/g in those with and without *H.influenzae* colonisation respectively (Table S9), although this difference was not statistically significant (p=0.08).

Patients colonised with *H.influenzae* and those with >1PPM had lower sputum eosinophil levels (medians 0.4% and 0.55% respectively) compared to patients without colonisation (median 1.0%; p=0.03 and 0.02 respectively, Figure S3B). Using >3% to define sputum eosinophilia, patients colonised with *H.influenzae* and >1PPM had fewer patients above this threshold compared to no colonisation (Table S9), although this difference was not significant (p*=*0.36). No significant differences in absolute sputum eosinophil counts were observed between groups. When comparing clinical characteristics of the groups, pack years were significantly lower in all groups compared to those with no colonisation (p=<0.01, Table S13). There were no differences in ICS use between groups.

*Blood cell counts*

In 226 patients with blood samples, there were no significant differences between the groups for absolute blood neutrophil or eosinophil cell counts (Figure S3C and Figure S3D) or eosinophil % (not shown). Blood neutrophil % was significantly increased in patients colonised with *H.influenzae* compared with no colonisation (medians: 67.7% and 63.4%, p=0.045, Figure S3E). The neutrophil-lymphocyte ratio was also significantly higher in *H.influenzae* colonised compared no colonisation group (medians: 3.44 and 2.56 respectively, p=0.01, Figure S3F). There were no significant differences in blood lymphocyte count between groups (data not shown).

***6 month results***

*Sputum cell counts*

Sputum DCC (*n*=66) showed that patients colonised with *H.influenzae* had significantly higher sputum neutrophil percentages compared to those with no colonisation; medians 86.6% and 68.4% respectively (p=0.048, Figure S4A). Absolute sputum neutrophil counts were also significantly higher in patients with *H.influenzae* colonisation compared to those without colonisation (16.4 and 1.38x10^6^/g, p<0.01, Table S11). There were no differences between other groups. Patients colonised with *H.influenzae* had lower sputum eosinophil levels compared to the no colonisation group (0.25% and 1.25% respectively, p*=*0.03, Figure S4B), with no significant differences for absolute counts.

*Blood cell counts*

In 103 patients with blood samples, there were no significant differences between groups for absolute blood eosinophil cell count, blood neutrophil % or NLR (Figure S4C-F).

**References**

1. Singh D, Agusti A, Anzueto A, Barnes PJ, Bourbeau J, Celli BR, et al. Global Strategy for the Diagnosis, Management, and Prevention of Chronic Obstructive Lung Disease: the GOLD science committee report 2019. European Respiratory Journal. 2019;53(5).

2. Taylor AE, Finney-Hayward TK, Quint JK, Thomas CM, Tudhope SJ, Wedzicha JA, et al. Defective macrophage phagocytosis of bacteria in COPD. European Respiratory Journal. 2010;35(5):1039-47.

3. Simpson JLea. COPD is characterized by increased detection of Haemophilus influenzae, Streptococcus pneumoniae and a deficiency of Bacillus species. Respirology. 2016;21(4):697-704.

4. Wang Z, Singh R, Miller BE, Tal-Singer R, Van Horn S, Tomsho L, et al. Sputum microbiome temporal variability and dysbiosis in chronic obstructive pulmonary disease exacerbations: an analysis of the COPDMAP study. Thorax. 2018;73(4):331-8.

5. Salter SJ, Cox MJ, Turek EM, Calus ST, Cookson WO, Moffatt MF, et al. Reagent and laboratory contamination can critically impact sequence-based microbiome analyses. BMC Biology. 2014;12:87.

**Results**

**
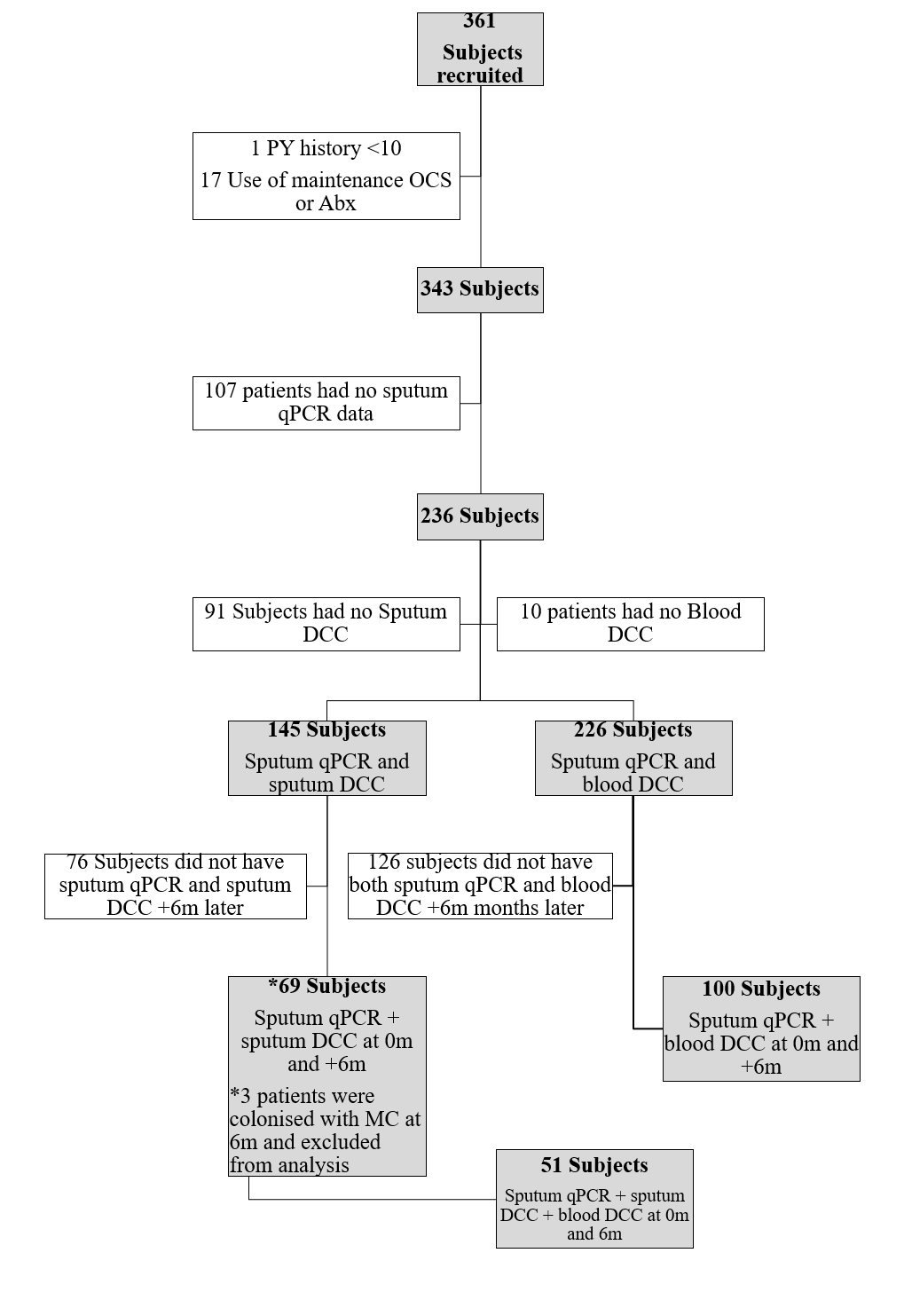
**

**Figure S1: Flow chart of subject selection process.** Flow chart for derivation of groups for analysis from 361 patients. Subjects were removed from analysis if; pack years (PY) <10, use of maintenance antibiotics (Abx) or oral corticosteroid (OCS) therapy and no sputum sample for quantitative (qPCR) analysis. Subjects were split into two groups determined by presence of sputum or blood differential cell count (DCC) data and further split for those with repeat data at 6 months follow up.

**
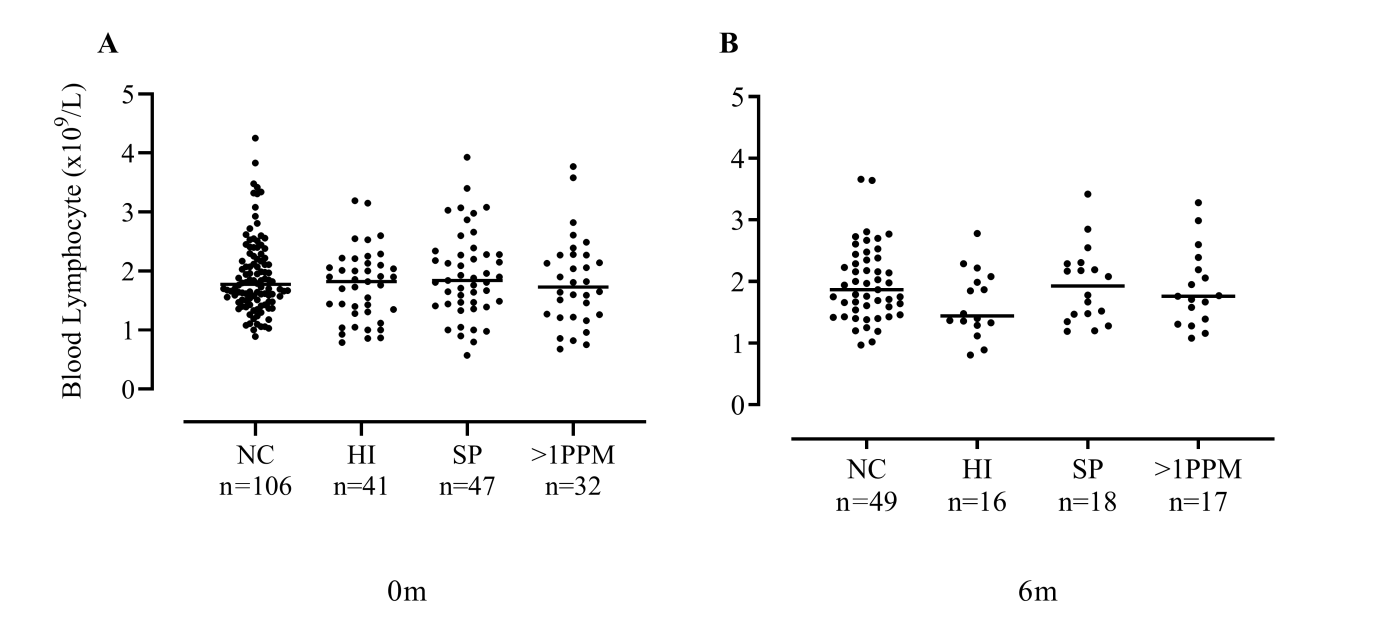
**

**Figure S2:** **The comparison of Blood Lymphocyte counts between patients colonised with different PPMs at 6 months post-baseline.** Patients were categorised into four groups based on bacterial load defined by genome copies/ml of ≥1x10^4^ no colonisation (NC), colonised with *Haemophilus influenzae* (HI), *Streptococcus pneumoniae* (SP) or >1 potentially pathogenic microorganism (PPM). Blood Lymphocyte count (n=226) are shown for each group at baseline (A) and 6 months (B). Statistical analysis was performed using Kruskal-Wallis and Mann-Whitney U as appropriate. Data represent individual patients with median.


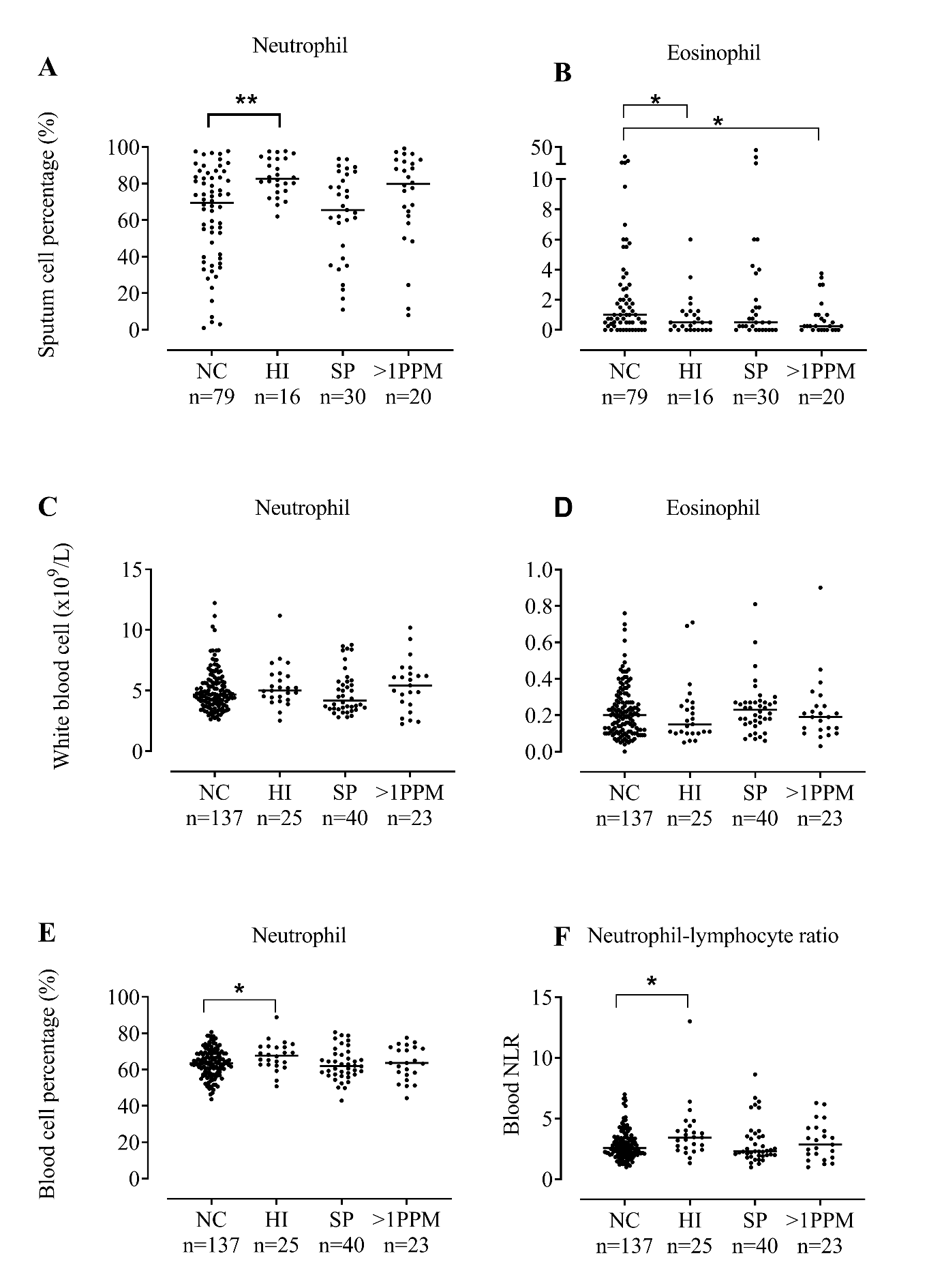


**Figure S3:** **The comparison of sputum and blood eosinophil and neutrophil counts between patients colonised with different PPMs**

Patients were categorised into four groups based on bacterial load defined by genome copies/ml of ≥1x10^6^; No colonisation (NC), colonised with *Haemophilus influenzae* (*HI*), *Streptococcus pneumoniae* (SP) or >1 potentially pathogenic microorganism (PPM). Sputum neutrophil % (n=145) (A), sputum eosinophil % (n=145) (B), blood neutrophil counts (n=226) (C), blood eosinophil counts (n=226) (D), blood neutrophil percentages (n=226) (E) and blood neutrophil-lymphocyte ratio (n=226) (F) are shown for each group. Statistical analysis was performed using Kruskal-Wallis and Mann-Whitney U adjusted for multiple comparisons. Data represent individual patients with median.

*, ** = significant difference to no colonisation group (*p<*0.05, <0.01 respectively).


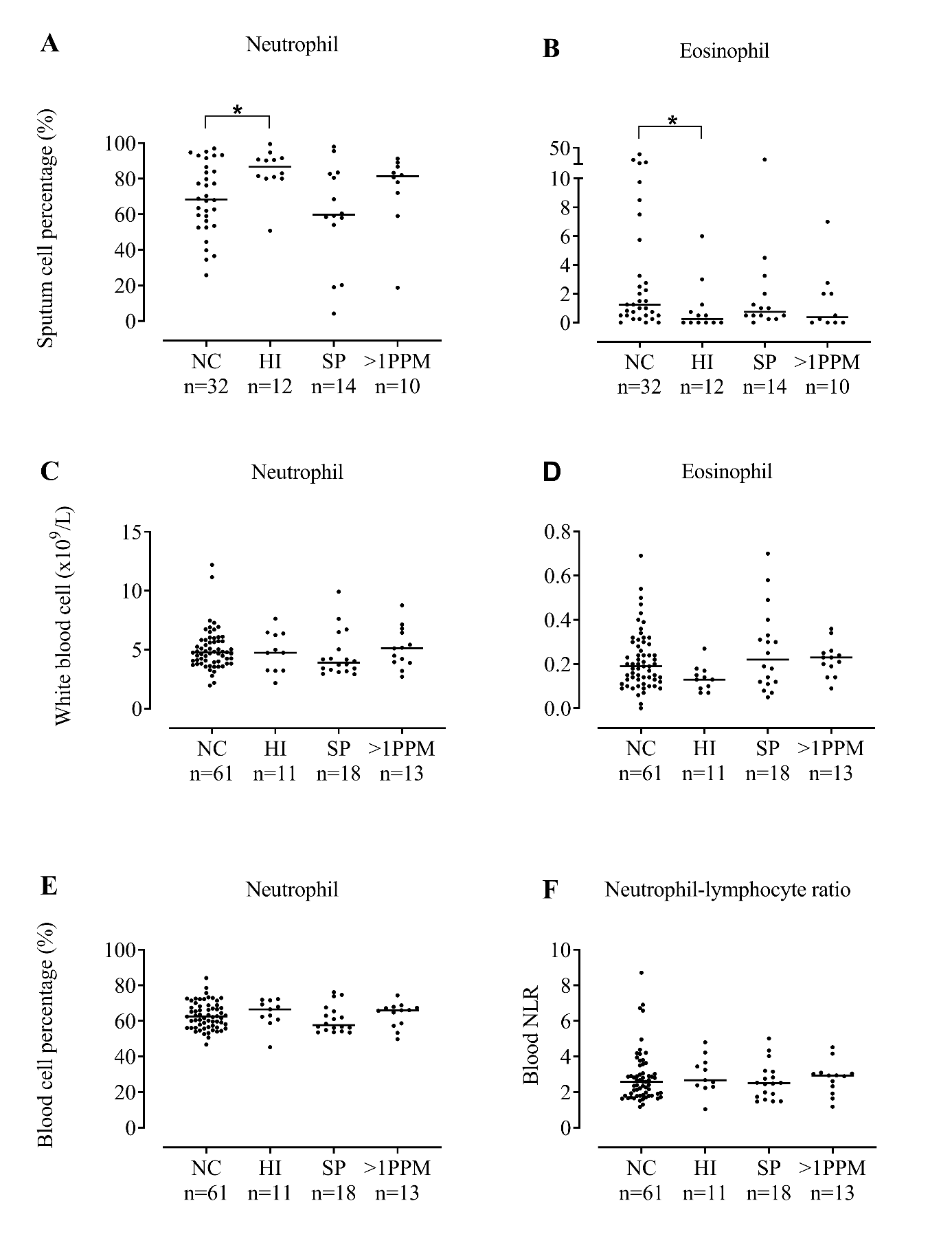


**Figure S4:** **The comparison of sputum and blood eosinophil and neutrophil counts between patients colonised with different PPMs at 6 months post-baseline.** Patients were categorised into four groups based on bacterial load defined by genome copies/ml of ≥1x10^6^; No colonisation (NC), colonised with *Haemophilus influenzae* (*HI*), *Streptococcus pneumoniae* (SP) or >1 potentially pathogenic microorganism (PPM). Sputum neutrophil % (n=68) (A), Sputum eosinophil % (n=68) (B). 1 patient was excluded due to colonisation with MC at 6 months. Blood neutrophil count (n=103) (C), Blood eosinophil count (n=103) (D) Blood neutrophil % (n=103) (E) and Blood neutrophil-lymphocyte ratio (n=103) (F). Statistical analysis was performed using Kruskal-Wallis and Mann-Whitney U adjusted for multiple comparisons. Data represent individual patients with median.

* = significant difference to no colonisation group (*p<*0.05 respectively)


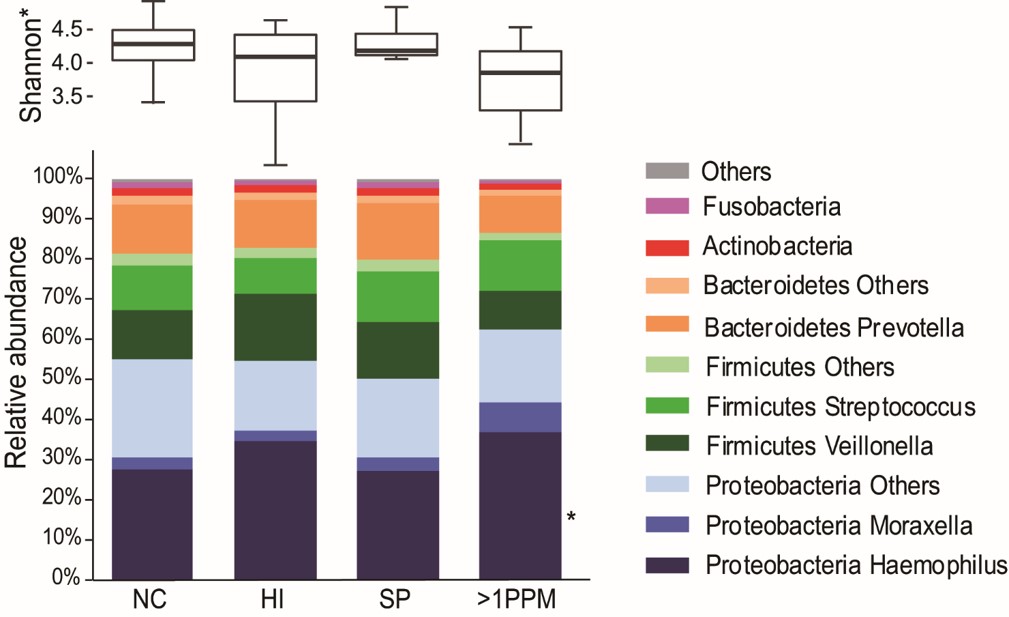


**Figure S5: Sputum microbiome profiles in COPD patients at 6 months**

A proportion of patients provided sufficient sample for 16s rRNA sequence analysis at 6 months. Shannon Diversity and relative abundance of major bacterial taxa and genera for PPM groups defined by genome copies per mL of ≥1x10^4^; No colonisation (NC), *Haemophilus influenzae* (*HI*), *Streptococcus pneumonia* (SP) and >1PPM n=73.

At 6 months *Haemophilus* abundance is increased in both HI and >1PPM groups, no other differences were observed between groups. Shannon diversity was significantly different between groups within the repeat cohort with a reduced alpha diversity in HI and >1PPM groups compared to no colonisation.

*, **, *** = significant difference to no colonisation group (FDR *p<*0.05, <0.01 and <0.001 respectively)





**Figure S6: Stability of *Haemophilus* presence over 6 months as defined by 16s rRNA Sequencing.** A proportion of patient with repeat measures of bacteria PCR and Sputum DCC provided sufficient sample for 16s rRNA sequencing. Scatter correlation of *Haemophilus* at baseline versus 6 months later (n=54). Statistical analysis was performed using Spearmans Rank order correlation.

**Tables**

**Table S1: Composition of >1PPM group at baseline.**

|  | *n* (%) |
| --- | --- |
| HI + SP + MC | 3 (8.3) |
| HI + SP | 21 (58.3) |
| HI + MC | 9 (25.0) |
| SP + MC | 3 (8.3) |

Patients were categorised into four groups based on bacterial load defined by genome copies/ml of ≥1x10^4^ no colonisation, colonised with *Haemophilus influenzae* (HI), *Streptococcus pneumoniae* (SP) or >1 potentially pathogenic microorganism (>1PPM) - including *Moraxella catarrhalis* (MC). The >1PPM group (n=36) contains the following combinations at baseline (HI + SP + MC (8.3%), HI + SP (58.3), HI+ MC (9%) and SP + MC (8.3%)).

**Table S2: Sputum inflammatory cell counts and qPCR for patients with frequent or infrequent exacerbations in the 12 months prior to baseline.**

|  | ***n*** | **Frequent exacerbator**  **(n=72)** | **Infrequent exacerbator**  **(n=73)** | **p-value** |
| --- | --- | --- | --- | --- |
| **Sputum total cell count x10^6^/g** | **112** | 3.89 [0.28- 84.56] | 3.58 [0.20-54.29] | 0.55 |
| **Sputum neutrophil %** | **145** | 75.73 [3.00-96.25] | 74.00 [1.00-96.25] | 0.89 |
| **Sputum macrophage %** | **145** | 15.70 [0.50-88.0] | 18.00 [2.00-87.00] | 0.68 |
| **Sputum eosinophil %** | **145** | 0.50 [0.00-26.00] | 1.00 [0.00-43.00] | 0.18 |
| **Sputum lymphocyte %** | **145** | 0.00 [0.00-2.00] | 0.00 [0.00-6.00] | 0.90 |
| **Sputum epithelial %** | **145** | 2.25 [0.00-89.25] | 1.88 [0.00-33.00] | 0.80 |
| **Sputum neutrophil cell count x10^6^/g** | **112** | 2.45 [0.02-82.44] | 2.56 [0.00-48.73] | >0.99 |
| **Sputum macrophage cell count x10^6^/g** | **112** | 0.69 [0.04-4.42] | 0.49 [0.04-2.23 | 0.45 |
| **Sputum eosinophil cell count x10^6^/g** | **112** | 0.02 [0.00-0.52] | 0.04 [0.00-2.04] | 0.12 |
| **Sputum lymphocyte cell count x10^6^/g** | **112** | 0.00 [0.00-0.18] | 0.00 [0.00-0.25] | 0.91 |
| **Sputum epithelial cell count x10^6^/g** | **112** | 0.14 [0.00-2.74] | 0.09 [0.00-2.32] | 0.09 |
| **Total SP load (copies/mL)** | **145** | 0.00 [0.00-1.47e+09] | 0.00 [0.00-9.5e+08] | 0.62 |
| **Total MC load (copies/mL)** | **145** | 0.00 [0.00-5.09e+09] | 0.00 [0.00-2.48e+09] | 0.67 |
| **Total HI load (copies/mL)** | **145** | 0.00 [0.00-1.57e+08] | 0.00 [0.00-1.59e+08] | 0.52 |
| **Total PPM load (copies/mL)** | **145** | 3.55e+04 [0.00-5.09e+09] | 6.96e+05 [0.00-2.64e+09] | 0.38 |
| **No colonisation (%)** | **145** | 47.21 | 38.36 | - |
| ***H.influenzae* (%)** | **145** | 16.67 | 19.18 | - |
| ***S.pneumoniae* (%)** | **145** | 18.06 | 24.66 | - |
| **>1PPM (%)** | **145** | 18.06 | 17.80 | - |

Patients were categorised into two groups based on the number of exacerbations recorded in the 12 months prior to baseline visits; Frequent (≥2 exacerbations) and Infrequent (<2 exacerbations. Statistical analysis was performed using Mann-Whitney U. Summaries are presented as percentages and Median [Range] and percentage (%) as appropriate (n=145^‡^).

^‡^33 patients had no absolute sputum cell counts available

**Table S3: Baseline sputum inflammatory cell counts for induced versus spontaneous sputum samples.**

|  | ***n*** | **Induced sample (n=25)** | **Spontaneous sample (n=118)** | | **p-value** |
| --- | --- | --- | --- | --- | --- |
| **Sputum total cell count x10^6^/g** | **112** | **6.59 [1.73-84.56]** | **3.48 [0.20-58.02]** | | **<0.01** |
| **Sputum neutrophil %** | **143** | 81.50 [22.90-97.75] | 73.92 [1.00-98.25] | | 0.13 |
| **Sputum macrophage %** | **143** | 15.40 [1.50-75.20] | 18.00 [0.50-88.50] | | 0.32 |
| **Sputum eosinophil %** | **143** | 0.52 [0.00-17.00] | 0.75 [0.00-43.00] | | 0.59 |
| **Sputum lymphocyte %** | **143** | 0.00 [0.00-1.50] | | 0.00 [0.00-6.00] | 0.37 |
| **Sputum epithelial %** | **143** | 1.00 [0.00-14.00] | | 2.50 [0.00-89.25] | 0.12 |
| **Sputum neutrophil cell count x10^6^/g** | **112** | **5.51 [0.66-82.44]** | | **2.27 [0.00-56.42]** | **<0.01** |
| **Sputum macrophage cell count x10^6^/g** | **112** | **1.02 [0.25-4.22]** | | **0.47 [0.04-4.42]** | **<0.01** |
| **Sputum eosinophil cell count x10^6^/g** | **112** | 0.08 [0.00-0.66] | | 0.02 [0.00-2.04] | 0.10 |
| **Sputum lymphocyte cell count x10^6^/g** | **112** | 0.00 [0.00-0.25] | | 0.00 [0.00-0.18] | 0.77 |
| **Sputum epithelial cell count x10^6^/g** | **112** | 0.08 [0.00-2.32] | | 0.12 [0.00-2.74] | 0.07 |
| **Total SP load (copies/mL)** | **143** | 0.00 [0.00-1.87e+08] | | 0.00 [0.00-1.47e+09] | 0.53 |
| **Total MC load (copies/mL)** | **143** | 0.00 [0.00-0.00] | | 0.00 [0.00-5.09e+09] | 0.22 |
| **Total HI load (copies/mL)** | **143** | 0.00 [0.00-1.36e+07] | | 0.00 [0.00-1.59e+08] | 0.89 |
| **Total PPM load (copies/mL)** | **143** | 0.00 [0.00-1.95e+08] | | 4.14e+05 [0.00-5.09e+09] | 0.12 |
| **No colonisation group (%)** | **143** | 56.00 | | 40.68 | - |
| ***H.influenzae* (%)** | **143** | 8.00 | | 18.64 | - |
| ***S.pneumoniae* (%)** | **143** | 16.00 | | 23.73 | - |
| **>1PPM (%)** | **143** | 20.00 | | 16.95 | - |

Patients were categorised into two groups based on the method of expectoration of sputum. Statistical analysis was performed using Mann-Whitney U. Summaries are presented as percentages and Median [Range] and percentage (%) as appropriate (n=143^‡^).

^‡^ 31 patients had no total or individual sputum cell counts available, as such n=112 are reported.

**Table S4: Baseline sputum inflammatory cell counts for different PPM groups.**

| **Baseline characteristic** | ***n*** | **NC** | **HI** | **SP** | **>1PPM** | ***p* value^§^** |
| --- | --- | --- | --- | --- | --- | --- |
| **Sputum total cell count x10^6^/g** | 112 | 2.81 [0.25-53.05] | ***9.68 [0.54-84.56]** | 3.07 [0.20-54.29] | 6.24 [0.28-31.82] | **0.02** |
| **Sputum Neutrophil %** | 145 | 69.5 [1-97.75] | ****82.63 [62-97.75]** | 65.5 [11-93.5] | 79.75 [8-99.25] | **<0.01** |
| **Sputum Eosinophil %** | 145 | 1.00 [0-27.50] | ***0.50 [0-6.00]** | 0.50 [0-43] | ***0.25 [0-3.75]** | **0.02** |
| **Sputum Lymphocyte %** | 145 | 0 [0-6.00] | 0.25 [0-3.00] | 0.25 [0-3.00] | 0 [0-2.00] | 0.45 |
| **Sputum Macrophage %** | 145 | 19.79 [1.5-88.5] | ****11.88 [1.50-28.00]** | 21.00 [3.25-87.00] | 14.83 [0.5-67.75] | **0.01** |
| **Sputum Epithelial Cells %** | 145 | 3.00 [0-89.25] | 1.00 [0-18.00] | 1.00 [0-40.50] | 1.63 [0-41.25] | 0.37 |
| **Sputum Neutrophil cell count x10^6^/g** | 112 | 1.71 [0.003-51.85] | ****8.59 [0.369-82.44]** | 2.42 [0.037-48.73] | 4.66 [0.032-30.95] | **0.01** |
| **Sputum Eosinophil cell count x10^6^/g** | 112 | 0.05 [0-0.66] | 0.02 [0-0.52] | 0.02 [0-2.04] | 0.02 [0-0.25] | 0.50 |
| **Sputum Lymphocyte cell count x10^6^/g** | 112 | 0 [0-0.18] | 0.01 [0-0.17] | 0.05 [0-0.06] | 0 [0-0.25] | 0.31 |
| **Sputum Macrophage cell count x10^6^/g** | 112 | 0.56 [0.04-2.26] | 0.58 [0.08-4.42] | 0.49 [0.1-3.26] | 0.67 [0.07-4.23] | 0.94 |
| **Sputum Epithelial cell count x10^6^/g** | 112 | 0.11 [0-2.35] | 0.17 [0-1.30] | 0.11 [0-2.74] | 0.15 [0-2.32] | 0.38 |
| **Sputum Eosinophil ≥3%** | 145 | 24.19 | 7.69 | 25.81 | 11.54 | 0.17 |

Patients were categorised into four groups based on bacterial load defined by genome copies/ml of ≥1x10^4^ no colonisation (NC), colonised with *Haemophilus influenzae* (HI), *Streptococcus pneumoniae* (SP) or >1 potentially pathogenic microorganism (>1PPM). Statistical analysis was performed using Kruskal-Wallis or Chi-Squared and Mann-Whitney U as appropriate. Summaries are presented as percentages and Median [Range] as appropriate (n = 145^‡^).

^§^ ANOVA, Kruskall-Wallis or Chi-squared tests were performed as appropriate.

*, ** = significant difference to no colonisation group (*p<*0.05, <0.01 respectively). Students t-test or Mann-Whitney U tests were performed as appropriate

^‡^33 patients had no absolute sputum cell counts available

**Table S5: Baseline Demographics of patients enrolled onto this study for different PPM groups.**

| **Baseline Characteristic** | **NC** | **HI** | **SP** | **>1PPM** | ***p***  ***value ^§^*** |
| --- | --- | --- | --- | --- | --- |
|  | n=108 | n=41 | n=47 | n=36 |  |
| **Gender (% Male)** | 70 | 76 | 75 | 86 | 0.32 |
| **Age** | 69.7 (7.8) | 70.1 (9.6) | 68.7 (8.3) | 69.7 (8.7) | 0.90 |
| **Smoking status (Current %)** | 25 | 32 | 43 | 51 | 0.05 |
| **Pack Years** | 47.0 [10-159] | 47.0 [10-140] | 52.0 [10-220] | 49.5 [10-138] | 0.24 |
| **BMI (kg/m2)** | 26.7[17.3-45.8] | ***24.3 [18.3-40.1]** | 27.3 [18.6-42.7] | 26.2 [18.0-41.9] | **0.05** |
| **Exacerbations (1 year period)** | 1 [1-15] | 1 [0-5] | 1 [0-7] | 1 [0-9] | 0.33 |
| **0** | 39 (36.1%) | 12 (29.3%) | 12 (25.5%) | 13 (36.1%) | - |
| **1** | 20 (18.5%) | 9 (22.0%) | 17 (36.2%) | 7 (19.4%) | - |
| **≥2** | 49 (45.4%) | 20 (48.8%) | 18 (38.3%) | 16 (44.4%) | - |
| **Post FEV1 (L)** | 1.5 (0.6) | 1.4 (0.6) | 1.4 (0.6) | 1.6 (0.5) | **0.04** |
| **Post FEV1 (%)** | 59.0 (19.1) | 51.9 (17.8) | 55.5 (20.3) | 59.6 (14.9) | 0.07 |
| **CAT** | 16.3 (8.9) | 17.9 (7.8) | 14.1 (10.3) | 18.4 (10.7) | 0.36 |
| **SGRQ-C (Total)** | 44.9 (17.8) | 49.3 (15.4) | ****50.1 (19.0)** | 46.5 (20.9) | **0.03** |
| **Chronic Bronchitis (%)** | 64 | 71 | 72 | 81 | 0.29 |
| **GOLD (%)** |  |  |  |  |  |
| **1** | 15.7 | 7.3 | 12.8 | 11.4 | - |
| **2** | 49.1 | 43.9 | 55.3 | 40.0 | - |
| **3** | 29.6 | 41.5 | 19.1 | 28.6 | - |
| **4** | 5.6 | 7.3 | 12.8 | 2.9 | - |
| **ICS use (%)** | 80 | 90 | 89 | 83 | 0.29 |
| **LABA Only (%)** | 3 | 2 | 4 | 0 | - |
| **LAMA Only (%)** | 5 | 2 | 4 | 14 | - |
| **ICS Only (%)** | 4 | 0 | 0 | 0 | - |
| **ICS + LABA (%)** | 16 | 7 | 15 | 11 | - |
| **ICS + LAMA (%)** | 1 | 0 | 2 | 3 | - |
| **LABA + LAMA (%)** | 4 | 2 | 0 | 0 | - |
| **Triple (%)** | 59 | 83 | 72 | 69 | - |
| **No inhaled medication (%)** | 9 | 2 | 2 | 3 | - |
| **CRP** | 3 [1-70] | 4.5 [1-16] | 3 [1-33] | 4 [1-58] | 0.52 |
| **Fibrinogen** | 3.1 [2.1-5.7] | 3.06 [1.9-7.0] | 3.11 [2.3-5.1] | 2.87 [2.1-7.7] | 0.91 |

Patients were categorised into four groups based on bacterial load defined by genome copies/ml of ≥1x10^4^ no colonisation (NC), colonised with *Haemophilus influenzae* (HI), *Streptococcus pneumoniae* (SP) or >1 potentially pathogenic microorganism (PPM). Statistical analysis was performed using Kruskal-Wallis or Chi-Squared and Mann-Whitney U as appropriate. Summaries are presented as percentages, Mean (SD) or Median [Range] as appropriate (n = 232^‡^). CRP values <1 were given an arbitrary value of 0.5.

^§^ ANOVA, Kruskall-Wallis or Chi-squared tests were performed as appropriate.

*, ** = significant difference to no colonisation group (*p<*0.05, <0.01 respectively). Students t-test or Mann-Whitney U tests were performed as appropriate

^‡^At baseline the following data were not available for; n=11 Saint Georges respiratory Questionnaire (SGRQ), n=8 Chronic obstructive pulmonary disease assessment test (CAT), n=93 fibrinogen, n=25 CRP and n=7 chronic bronchitis questionnaires.

**Table S6: Baseline blood inflammatory cell counts for different PPM groups.**

| **Baseline characteristic** | ***n*** | **NC** | **HI** | **SP** | **>1PPM** | ***p* value^§^** |
| --- | --- | --- | --- | --- | --- | --- |
| **Blood Neutrophil %** | 226 | 63.51 [43.67-78.66] | ***67.1 [50.5-88.81]** | 61.83 [42.94-80.38] | 63.65 [44.31-78.9] | 0.06 |
| **Blood Neutrophil x10^9^/L** | 226 | 4.68 [2.62-12.23] | 4.99 [2.54-11.19] | 4.19 [2.81-8.79] | 4.95 [2.26-10.2] | 0.17 |
| **Blood Eosinophil %** | 226 | 2.52 [0-11.18] | 2.05 [0.4-8.66] | 2.72 [0.67-11.91] | 2.76 [0.23-8.26] | 0.71 |
| **Blood Eosinophil x10^9^/L** | 226 | 0.2 [0-0.76] | 0.18 [0.05-0.71] | 0.21 [0.04-0.81] | 0.2 [0.03-0.9] | 0.96 |
| **Neutrophil Lymphocyte Ratio** | 226 | 2.56 [0.99-6.99] | ****3.32 [1.27-13.01]** | 2.29 [0.98-8.63] | 2.87 [0.99-6.7] | **0.02** |

Patients were categorised into four groups based on bacterial load defined by genome copies/ml of ≥1x10^4^ no colonisation (NC), colonised with *Haemophilus influenzae* (HI), *Streptococcus pneumoniae* (SP) or >1 potentially pathogenic microorganism (PPM). Statistical analysis was performed using Kruskal-Wallis or Chi-Squared and Mann-Whitney U as appropriate. Summaries are presented as percentages and Median [Range] as appropriate (n = 225).

^§^ ANOVA or Kruskall-Wallis tests were performed as appropriate.

*, ** = significant difference to no colonisation group (*p<*0.05, <0.01 respectively). Students t-test or Mann-Whitney U tests were performed as appropriate

**Table S7: Sputum inflammatory cell counts for different PPM groups at 6 months.**

| **6m Characteristics** | *n* | **NC** | **HI** | **SP** | **>1PPM** | ***p* value^§^** |
| --- | --- | --- | --- | --- | --- | --- |
| **Sputum total cell count x10^6^/g** | 60 | 2.87[0.33-16.25] | 14.84 [0.67-36.34] | 3.06 [0.27-24] | 1.84 [0.42-5.57] | 0.01 |
| **Sputum Neutrophil %** | 66 | 69.54 [25.75-94.75] | ***86.63 [56.25-95.25]** | 64.13 [19-98] | 75 [4.25-91.25] | 0.06 |
| **Sputum Eosinophil %** | 66 | 2.13 [0-34] | ***0.38 [0-14]** | 0.63 [0-20.75] | 0.75 [0-7.0] | **0.02** |
| **Sputum Lymphocyte %** | 66 | 0 [0-0.75] | 0 [0-0.75] | 0 [0-1.25] | 0 [0-0.25] | 0.73 |
| **Sputum Macrophage %** | 66 | 18.50 [1.75-41.75] | 6.00 [1.50-30.75] | 16.13 [1.25-55.5] | 16.25 [6.5-84.25] | 0.06 |
| **Sputum Epithelial Cells %** | 66 | 2.13 [0-34] | 2.13 [0-16.75] | 6.88 [0.75-56.5] | 3 [0-58.75] | 0.06 |
| **Sputum Neutrophil cell count x10^6^/g** | 60 | 1.45 [0.15-12.39] | ****12.89 [0.40-32.98]** | 1.73 [0.05-23.52] | 1.11 [0.08 -5.08] | **<0.01** |
| **Sputum Eosinophil cell count x10^6^/g** | 60 | 0.07 [0-1.512] | 0.04 [0-2.16] | 0.03 [0-0.67] | 0.02 [0-0.25] | 0.33 |
| **Sputum Lymphocyte cell count x10^6^/g** | 60 | 0 [0-0.0231] | 0 [0-0.2726] | 0 [0-0.0602] | 0 [0-0.03] | 0.93 |
| **Sputum Macrophage cell count x10^6^/g** | 60 | 0.31 [0.1073-2.965] | 0.63 [0.1005-3.777] | 0.4854 [0.0621 -1.504] | 0.35 [0.09-2.49] | 0.48 |
| **Sputum Epithelial cell count x10^6^/g** | 60 | 0.12 [0.01-0.61] | 0.24 [0.08-3.00] | 0.21 [0.04-2.74] | 0.13 [0.02-0.47] | 0.08 |
| **Sputum Eosinophil ≥3%** | 66 | 63.64 | 14.29 | 25 | 12.5 | **<0.01** |

Patients were categorised into four groups based on bacterial load defined by genome copies/ml of ≥1x10^4^ no colonisation (NC), colonised with *Haemophilus influenzae* (HI), *Streptococcus pneumoniae* (SP) or >1 potentially pathogenic microorganism (PPM). Statistical analysis was performed using Kruskal-Wallis or Chi-Squared and Mann-Whitney U as appropriate. Summaries are presented as percentages and Median [Range] as appropriately for sputum cell counts (n = 66^‡^).

^§^ ANOVA, Kruskall-Wallis or Chi-squared tests were performed as appropriate.

*, ** = significant difference to no colonisation group (*p<*0.05, <0.01 respectively). Students t-test or Mann-Whitney U tests were performed as appropriate

^‡^ 6 patients had no absolute sputum cell counts available

**Table S8: Blood inflammatory cell counts for different PPM groups at 6 months.**

| **6m Characteristics** | *n* | **NC** | **HI** | **SP** | **>1PPM** | ***p* value^§^** |
| --- | --- | --- | --- | --- | --- | --- |
| **Blood Neutrophil %** | 100 | 62.67 [50.64-84.14] | 66.78 [45.21-75.68] | 56.48 [46.67-76.2] | 65.94 [49.74-74.67] | 0.05 |
| **Blood Neutrophil x10^9^/L** | 100 | 4.81 [2.19-12.2] | 4.71 [2.17-7.63] | ***3.73 [1.96-9.93]** | 4.81 [2.7-8.77] | 0.10 |
| **Blood Eosinophil %** | 100 | 2.58 [0-7.62] | 1.84 [0.93-4.91] | 2.80 [0.5-11.15] | 2.61 [1.10-6] | 0.32 |
| **Blood Eosinophil x10^9^/L** | 100 | 0.2 [0-0.69] | 0.14 [0.07-0.3] | 0.22 [0.05-0.7] | 0.21 [0.09-0.36] | 0.16 |
| **Neutrophil Lymphocyte Ratio** | 100 | 2.67 [1.3-8.71] | 2.63 [1.04-6.91] | 2.1 [1.17-5.01] | 2.94 [1.18-4.52] | 0.12 |

Patients were categorised into four groups based on bacterial load defined by genome copies/ml of ≥1x10^4^ no colonisation (NC), colonised with *Haemophilus influenzae* (HI), *Streptococcus pneumoniae* (SP) or >1 potentially pathogenic microorganism (PPM). Statistical analysis was performed using Kruskal-Wallis and Mann-Whitney U as appropriate. Summaries are presented as percentages and Median [Range] as appropriately blood cell counts (n=100).

^§^ ANOVA or Kruskall-Wallis tests were performed as appropriate.

*, ** = significant difference to no colonisation group (*p<*0.05, <0.01 respectively). Students t-test or Mann-Whitney U tests were performed as appropriate

**Table S9: Baseline sputum inflammatory cell counts for different PPM groups, x10^6^ copies/ml to detect PPMs.**

| **Baseline characteristic** | ***n*** | **NC** | **HI** | **SP** | **>1PPM** | ***ANOVA***  ***p-*value^§^** |
| --- | --- | --- | --- | --- | --- | --- |
| **Sputum total cell count x106/g** | 113 | 2.96 [0.22-53.05] | 8.70 [0.28-84.56] | 2.62 [0.2-54.29] | 6.59 [1.02-31.82] | 0.08 |
| **Sputum Neutrophil (%)** | 145 | 72.0 [1.0-97.8] | ****83.4 [11.5-97.8]** | 67.5 [8.0-99.3] | 82.1 [24.5-97.3] | **<0.01** |
| **Sputum Eosinophil (%)** | 145 | 1.0 [0-27.5] | *0.4 [0-6.0] | 0.25 [0-43.0] | *0.55 [0-3.75] | **0.02** |
| **Sputum Lymphocyte (%)** | 145 | 0.1 [0.0-6.0] | 0 [0.0-2.0] | 0.0 [0.0-1.0] | 0.0 [0.0-2.0] | 0.21 |
| **Sputum Macrophage (%)** | 145 | 18.25 [1.5-88.50] | ***8.79 [1.5-45.75]** | 19.13 [0.5-87.0] | 11.37 [2.0-67.75] | 0.02 |
| **Sputum Epithelial Cells (%)** | 145 | 3.0 [0.0-89.25] | 1.0 [0.0-41.25] | 1.00 [0.0-40.50] | 1.25 [0.0-14.00] | 0.29 |
| **Sputum Neutrophil cell count x10^6^/g** | 113 | 2.03 [0.0-51.85] | 8.26 [0.03-82.44] | 2.45 [0.05-48.73] | 5.51 [0.33-30.95] | 0.07 |
| **Sputum Eosinophil cell count x10^6^/g** | 113 | 0.03 [0.0-0.50] | 0.04 [0.0-0.52] | 0.02 [0.0-2.04] | 0.04 [0.0-0.25] | 0.90 |
| **Sputum Lymphocyte cell count x10^6^/g** | 113 | 0.0 [0.0-0.18] | 0.0 [0.0-0.09] | 0.0 [0.0-0.03] | 0.0 [0.0-0.25] | 0.29 |
| **Sputum Macrophage cell count x10^6^/g** | 113 | 0.56 [0.04-2.26] | 0.45 [0.08-4.42] | 0.47 [0.07 -3.26] | 0.69 [0.08-4.23] | 0.64 |
| **Sputum Epithelial cell count x10^6^/g** | 113 | 0.11 [0.0-2.74] | 0.18 [0.0-1.3] | 0.1 [0.0-0.48] | 0.18 [0.01-2.32] | 0.53 |
| **Sputum Eosinophil ≥3%** | 145 | 18 (22.78%) | 2 (12.5%) | 6 (35.29%) | 3 (15%) | 0.36 |

Patients were categorised into four groups based on bacterial load defined by genome copies/ml of ≥1x10^6^ no colonisation (NC), colonised with *Haemophilus influenzae* (HI), *Streptococcus pneumoniae* (SP) or >1 potentially pathogenic microorganism (PPM). Statistical analysis was performed using Kruskal-Wallis or Chi-Squared and Mann-Whitney U as appropriate. Summaries are presented as percentages and Median [Range] as appropriate (n = 145^‡^).

^§^ ANOVA or Kruskall-Wallis tests were performed as appropriate.

*, ** = significant difference to no colonisation group (*p<*0.05, <0.01 respectively). Students t-test or Mann-Whitney U tests were performed as appropriate

^‡^32 patients had no absolute sputum cell counts available

**Table S10: Baseline blood inflammatory cell counts for different PPM groups, x10^6^ copies/ml to detect PPMs.**

| **Baseline characteristic** | ***n*** | **NC** | **HI** | **SP** | **>1PPM** | ***ANOVA***  ***p-*value^§^** |
| --- | --- | --- | --- | --- | --- | --- |
| **Blood Neutrophil (%)** | 226 | 63.58 [43.67-80.68] | ***67.74 [50.80-88.81]** | 61.97 [42.94-80.57] | 63.65 [44.31-77.57] | 0.12 |
| **Blood Neutrophil (x10^9^/L)** | 226 | 4.68 [2.62-11.16] | 5.01 [2.54-11.19] | 4.2 [2.81-8.79] | 5.44 [2.26-10.20] | 0.18 |
| **Blood Eosinophil (%)** | 226 | 2.5 [0-11.2] | 1.97 [0.4-8.66] | 2.96 [0.76-11.9] | 2.66 [0.23-8.26] | 0.41 |
| **Blood Eosinophill (x10^9^/L)** | 226 | 0.2 [0-0.76] | 0.15 [0.05-0.71] | 0.23 [0.06-0.81] | 0.19 [0.03-0.9] | 0.91 |
| **Neutrophil Lymphocyte Ratio** | 226 | 2.56 [0.99-6.99] | ***3.44 [1.33-13.01]** | 2.30 [0.98-8.63] | 2.86 [0.99-6.29] | 0.05 |

Patients were categorised into four groups based on bacterial load defined by genome copies/ml of ≥1x10^6^ no colonisation (NC), colonised with *Haemophilus influenzae* (HI), *Streptococcus pneumoniae* (SP) or >1 potentially pathogenic microorganism (PPM). Statistical analysis was performed using Kruskal-Wallis or Chi-Squared and Mann-Whitney U as appropriate. Summaries are presented as percentages and Median [Range] as appropriate (n = 226).

^§^ ANOVA or Kruskall-Wallis tests were performed as appropriate.

* = significant difference to no colonisation group (*p<*0.05). Students t-test or Mann-Whitney U tests were performed as appropriate

**Table S11: Sputum inflammatory cell counts for different PPM groups at 6 months, x10^6^ copies/ml to detect PPMs.**

| **6m characteristic** | ***n*** | **NC** | **HI** | **SP** | **>1PPM** | ***ANOVA***  ***p-*value^§^** |
| --- | --- | --- | --- | --- | --- | --- |
| **Sputum total cell count x106/g** | 61 | 2.33 [0.33-51.32] | ****19.83 [1.08-36.34]** | 2.91 [0.27-24.00] | 1.68 [0.42-5.57] | **<0.01** |
| **Sputum Neutrophil (%)** | 68 | 68.38 [25.75-97.00] | ***86.63 [50.73-99.50]** | 59.88 [4.25-98.00] | 81.38 [18.75-91.25] | 0.07 |
| **Sputum Eosinophil (%)** | 68 | 1.25 [0.0-34.0] | ***0.25 [0.0-6.00]** | 0.75 [0-7.00] | 0.38 [0.00-7.0] | 0.06 |
| **Sputum Lymphocyte (%)** | 68 | 0.0 [0.0-0.75] | 0.0 [0.0-0.75] | 0.13 [0.0-1.50] | 0.0 [0.0-1.00] | 0.64 |
| **Sputum Macrophage (%)** | 68 | 16.88 [0.50-42.22] | 63.00 [0.50-39.50] | 20 [1.25-84.25] | 14.50 [6.5-28.00] | 0.14 |
| **Sputum Epithelial Cells (%)** | 68 | 4.13 [0.0-38.75] | 1.13 [0.0-16.75] | 7.5 [0.75-56.50] | 1.88 [0.0-58.75] | **0.02** |
| **Sputum Neutrophil cell count x10^6^/g** | 61 | 1.38 [0.15-49.78] | ****16.44 [0.55-32.98]** | 1.60 [0.05-23.52] | 1.33 [0.08-5.08] | **<0.01** |
| **Sputum Eosinophil cell count x10^6^/g** | 61 | 0.04 [0.0-1.51] | 0.02 [0.0-2.16] | 0.02 [0.0-0.67] | 0.01 [0.0-0.25] | 0.52 |
| **Sputum Lymphocyte cell count x10^6^/g** | 61 | 0.0 [0.0-0.02] | 0.0 [0.0-0.27] | 0.0 [0.0-0.06] | 0.0 [0.0-0.02] | 0.69 |
| **Sputum Macrophage cell count x10^6^/g** | 61 | 0.31 [0.1-2.96] | 0.63 [0.11-3.78] | 0.49 [0.06-2.49] | 0.33 [0.09-0.51] | 0.19 |
| **Sputum Epithelial cell count x10^6^/g** | 61 | 0.14 [0.02-1.03] | 0.21 [0.0-3.00] | 0.2 [0.04-0.46] | 0.05 [0.02-0.29] | 0.16 |
| **Sputum Eosinophil ≥3%** | 68 | 9 (28.13%) | 2 (16.67%) | 3 (21.43%) | 1 (10%) | 0.63 |

Patients were categorised into four groups based on bacterial load defined by genome copies/ml of ≥1x10^6^ no colonisation (NC), colonised with *Haemophilus influenzae* (HI), *Streptococcus pneumoniae* (SP) or >1 potentially pathogenic microorganism (PPM). Statistical analysis was performed using Kruskal-Wallis or Chi-Squared and Mann-Whitney U as appropriate. Summaries are presented as percentages and Median [Range] as appropriately for sputum cell counts (n = 68^‡^).

^§^ ANOVA, Kruskall-Wallis or Chi-squared tests were performed as appropriate.

*, ** = significant difference to no colonisation group (*p<*0.05, <0.01 respectively). Students t-test or Mann-Whitney U tests were performed as appropriate

^‡^ 7 patients had no absolute sputum cell counts available

**Table S12: Blood inflammatory cell counts for different PPM groups at 6 months, x10^6^ copies/ml to detect PPMs.**

| **6m characteristic** | ***n*** | **NC** | **HI** | **SP** | **>1PPM** | ***ANOVA p-*value^§^** |
| --- | --- | --- | --- | --- | --- | --- |
| **Blood Neutrophil (%)** | 103 | 62.46 [46.67-84.14] | 66.53 [45.21-72.25] | 57.63 [53.45-76.20] | 65.94 [49.74-74.3] | 0.37 |
| **Blood Neutrophil (x10^9^/L)** | 103 | 4.76 [1.96-12.20] | 4.74 [2.17-7.63] | 3.89 [2.92-9.93] | 5.12 [2.70-8.77] | 0.33 |
| **Blood Eosinophil (%)** | 103 | 2.55 [0.0-7.40] | 1.67 [0.93-4.91] | 2.77 [0.5-11.15] | 2.99 [1.10-6.00] | 0.16 |
| **Blood Eosinophill (x10^9^/L)** | 103 | 0.19 [0.0-0.69] | 0.13 [0.07-0.27] | 0.22 [0.05-0.70] | 0.23 [0.09-0.36] | 0.09 |
| **Neutrophil Lymphocyte Ratio** | 103 | 2.58 [1.17-8.71] | 2.67 [1.04-4.80] | 2.5 [1.47-5.01] | 2.93 [1.18-4.52] | 0.62 |

Patients were categorised into four groups based on bacterial load defined by genome copies/ml of ≥1x10^6^ no colonisation (NC), colonised with *Haemophilus influenzae* (HI), *Streptococcus pneumoniae* (SP) or >1 potentially pathogenic microorganism (PPM). Statistical analysis was performed using Kruskal-Wallis and Mann-Whitney U as appropriate. Summaries are presented as percentages and Median [Range] as appropriately for blood cell counts (n=103).

^§^ ANOVA or Kruskall-Wallis tests were performed as appropriate.

**Table S13: Baseline demographics, sputum and blood inflammatory cell counts for different PPM groups, x10^6^ copies/ml to detect PPMs.**

|  | **NC** | **HI** | **SP** | **>1PPM** |  |
| --- | --- | --- | --- | --- | --- |
| **Characteristic** | ***n=139*** | **n=25** | **n=41** | **n=26** | ***p value ^§^*** |
| **Gender (% Male)** | 74.1 | 76 | 75.6 | 73.1 | 0.99 |
| **Age** | 69.7 (8.0) | 69.5 (8.3) | 70.7 (8.6) | 68.0 (10.3) | 0.65 |
| **Smoking status (Current %)** | 35.3 | 32 | 41.5 | 19.2 | 0.30 |
| **Pack Years** | 49 [10-201] | ****48.0 [11.0-135.00]** | ****47 [10-220]** | ****48.0 [10.-159.0]** | **<0.01** |
| **BMI (kg/m2)** | 26.5 [18.0-45.8] | 28.2 [20.7-36.3] | 24.5 [19.3-42.0] | 27.0 [17.3-42.9] | 0.22 |
| **Exacerbations (1 year period)** | 1 [0-15] | 1 [0-12] | 1 [0-13] | 1 [0-5] | 0.89 |
| **Post FEV1 (L)** | 1.5 (0.5) | 1.7 (0.6) | 1.4 (0.5) | 1.4 (0.6) | **0.047** |
| **Post FEV1 (%)** | 58.6 (18.6) | 63.9 (18.6) | 55.8 (19.0) | 51.3 (16.6) | 0.09 |
| **CAT** | 17.1 (8.5) | 14.9 (10.0) | 15.1 (9.1) | 17.8 (10.3) | 0.44 |
| **SGRQ-C (Total)** | 48.8 (16.9) | 42.1 (18.0) | 42.9 (21.8) | 51.2 (17.2) | 0.20 |
| **GOLD (%)** |  |  |  |  |  |
| **1** | 16.5 | 20 | 7.3 | 3.8 | - |
| **2** | 51.1 | 56 | 43.9 | 53.9 | - |
| **3** | 26.6 | 16 | 41.5 | 30.8 | - |
| **4** | 5.8 | 8 | 7.3 | 11.5 | - |
| **ICS Use (%)** | 82.0 | 76.0 | 87.8 | 92.3 | 0.35 |
| **LABA Only (%)** | 3.6 | 0 | 0 | 0 | - |
| **LAMA Only (%)** | 6.5 | 0 | 2.4 | 3.9 | - |
| **ICS Only (%)** | 1.4 | 8 | 0 | 0 | - |
| **ICS + LABA (%)** | 14.4 | 20 | 4.9 | 11.5 | - |
| **ICS + LAMA (%)** | 0.7 | 4 | 2.4 | 0 | - |
| **LABA + LAMA (%)** | 2.9 | 0 | 0 | 0 | - |
| **Triple (%)** | 65.5 | 44 | 80.5 | 80.8 | - |
| **No inhaled Medication (%)** | 4.3 | 24 | 2.4 | 0 | - |
| **Fibrinogen** | 3.0 [1.9-7.7] | 3.1 [2.3-5.1] | 2.8 [2.2-5.3] | 3.3 [2.2-5.5] | 0.62 |
| **CRP** | 3.0 [1.0-157.0] | 3.0 [1.0-70.0] | 3 [1-15] | 5.0 [1.0-13.0] | 0.5906 |

Patients were categorised into four groups based on bacterial load defined by genome copies/ml of ≥1x10^6^ no colonisation (NC), colonised with *Haemophilus influenzae* (HI), *Streptococcus pneumoniae* (SP) or >1 potentially pathogenic microorganism (PPM). Statistical analysis was performed using Kruskal-Wallis or Chi-Squared and Mann-Whitney U as appropriate. Summaries are presented as percentages, Mean (SD) or Median [Range] as appropriate (n = 231^‡^). CRP values <1 were given an arbitrary value of 0.5.

^§^ ANOVA, Kruskall-Wallis or Chi-squared tests were performed as appropriate.

** = significant difference to no colonisation group (*p*<0.01). Students t-test or Mann-Whitney U tests were performed as appropriate

^‡^At baseline the following data were not available for; n=9 Saint Georges respiratory Questionnaire (SGRQ), n=6 Chronic obstructive pulmonary disease assessment test (CAT), n=98 fibrinogen and n=36 CRP.

**Table S14: Baseline demographics for different PPM groups.**

|  | **NO-NO** | **HI-HI** | ***P value ^§^*** |
| --- | --- | --- | --- |
| **Characteristic** | n=30 | n=9 |  |
| **Gender (% Male)** | 76 | 78 | >0.99 |
| **Age** | 69.7 (6.5) | 68.8 (9.8) | 0.74 |
| **Smoking status (Current %)** | 20 | 11 | >0.99 |
| **Pack Years** | 47 [18-113] | 35 [22-72] | 0.19 |
| **BMI (kg/m2)** | 26.97 (4.32) | 25.79 (2.68) | 0.45 |
| **Exacerbations (1 year period) at baseline** | 1 [0-6] | 2 [0-5] | 0.25 |
| **Exacerbations between visits** | 0 [0-2] | 0 [0-2] | 0.45 |
| **Exacerbations (1 year period) at 12m** | 1 [0-5] | 2 [0-4] | 0.12 |
| **Post FEV1 (L)** | 1.7 [0.4-3.4] | 0.9 [0.9-2.4] | 0.09 |
| **Post FEV1 (%)** | 61.2 (18.6) | ***46.0 (17.6)** | **0.04** |
| **CAT** | 16.6 (6.3) | 19.4 (5.8) | 0.24 |
| **SGRQ-C (Total)** | 46.2 (17.7) | 46.8 (11.8) | 0.93 |
| **Chronic Bronchitis (%)** | 79 | 78 | >0.99 |
| **ICS use (%)** | 80 | 89 | >0.99 |
| **LABA Only (%)** | 0 | 0 | - |
| **LAMA Only (%)** | 7 | 11 | - |
| **ICS Only (%)** | 7 | 0 | - |
| **ICS + LABA (%)** | 7 | 0 | - |
| **ICS + LAMA (%)** | 0 | 0 | - |
| **LABA + LAMA (%)** | 0 | 0 | - |
| **Triple (%)** | 67 | 89 | - |
| **No inhaled medication (%)** | 13 | 0 | - |
| **CRP** | 3 [1-28] | 3 [1-11] | 0.13 |

Patients were categorised into two groups based on bacterial load defined by genome copies/ml of ≥1x10^4^ at baseline and 6 months later; NO-NO (No colonisation at either visit) and HI-HI (Presence of *Haemophilus influenzae* at both visits). Statistical analysis was performed using Chi-squared and Mann-Whitney U as appropriate. Summaries are presented as percentages, Mean (SD) and Median [Range] as appropriate (n = 39 ^‡^).

*, ** = significant difference (*p<*0.05 and <0.01 respectively)

***^§^*** Students t-test, Mann-Whitney U or Chi-squared tests were performed as appropriate.

^‡^ The following data were not available for; n=2 exacerbation data (between visits), n=6 exacerbation data (1 year period) at 12 months and n=5 CRP

**Table S15: Sputum and blood inflammatory cell counts for different PPM groups.**

|  | **NO-NO** | **HI-HI** | ***P value ^§^*** |
| --- | --- | --- | --- |
| **Characteristic** | n=30 | n=9 |  |
| **Sputum total cell count x10^6^/g** | 3.01 [0.31-22.15] | ***9.36 [1.02-84.56]** | **0.04** |
| **Sputum Neutrophil (%)** | 68.13 [1-96.25] | ***83.53 [62-97.75]** | **0.01** |
| **Sputum Eosinophil (%)** | 2.13 [0-17] | ***0.25 [0-3.5]** | **0.02** |
| **Sputum Lymphocyte (%)** | 0.05 [0-6] | 0 [0-1] | 0.53 |
| **Sputum Macrophage (%)** | 18.67 [2-85.5] | ***8.82 [1.5-28]** | **0.02** |
| **Sputum Epithelial Cells (%)** | 4.18 [0-31] | 2 [0-18] | 0.42 |
| **Sputum Neutrophil cell count x10^6^/g** | 1.87 [0-19.88] | ***9.15 [0.63-82.44]** | **0.03** |
| **Sputum Eosinophil cell count x10^6^/g** | 0.08 [0-0.66] | 0.01 [0-0.42] | 0.38 |
| **Sputum Lymphocyte cell count x10^6^/g** | 0 [0-0.18] | 0 [0-0.09] | 0.86 |
| **Sputum Macrophage cell count x10^6^/g** | 0.51 [0.14-2.16] | 0.34 [0.14-4.42] | 0.88 |
| **Sputum Epithelial cell count x10^6^/g** | 0.1 [0-0.41] | 0.22 [0-1.3] | 0.21 |
| **Sputum Eosinophil ≥3% (%)** | 40 | 11 | 0.12 |
| **Sputum Eosinophil ≥2% (%)** | 60 | ****11** | **0.02** |

Patients were categorised into two groups based on bacterial load defined by genome copies/ml of ≥1x10^4^ at baseline and 6 months later; NO-NO (No colonisation at either visit) and HI-HI (Presence of *Haemophilus influenzae* at both visits). Statistical analysis was performed using Chi-squared and Mann-Whitney U as appropriate. Summaries are presented as percentages, Mean (SD) and Median [Range] as appropriate (n = 39 ^‡^).

*, ** = significant difference (*p<*0.05 and <0.01 respectively)

***^§^*** Students t-test, Mann-Whitney U or Chi-squared tests were performed as appropriate.

^‡^ The following data were not available; 14 patients had no absolute sputum cell counts available, 10 patients had no sputum cell count % available.

**Table S16: Baseline demographics for different PPM groups, including those with *H.influenzae* + ≥1 PPM.**

|  | **NO-NO** | **HI-HI** | ***P value*** |
| --- | --- | --- | --- |
| **Characteristic** | n=30 | n=25 |  |
| **Gender (% Male)** | 76 | 80 | >0.99 |
| **Age** | 69.7 (6.5) | 69.5 (9.0) | 0.92 |
| **Smoking status (Current %)** | 20 | 32 | 0.36 |
| **Pack Years** | 47 [18-113] | 41 [10-140] | 0.26 |
| **BMI (kg/m2)** | 26.97 (4.32) | 26.23 (4.98) | 0.56 |
| **Exacerbations (1 year period) at baseline** | 1 [0-6] | 1 [0-9] | 0.32 |
| **Exacerbations between visits** | 0 [0-2] | 0 [0-3] | 0.67 |
| **Exacerbations (1 year period) at 12m** | 1 [0-5] | 1 [0-7] | 0.90 |
| **Post FEV1 (L)** | 1.7 [0.4-3.4] | ***1.2 [0.4-2.4]** | **0.03** |
| **Post FEV1 (%)** | 61.2 (18.6) | ***49.3 (15.7)** | **0.01** |
| **CAT** | 16.6 (6.3) | 19.4 (7.0) | 0.13 |
| **SGRQ-C (Total)** | 46.2 (17.7) | 45.8 (17.1) | 0.93 |
| **Chronic Bronchitis (%)** | 79 | 84 | 0.74 |
| **ICS use (%)** | 80 | 92 | 0.27 |
| **LABA Only (%)** | 0 | 0 | - |
| **LAMA Only (%)** | 7 | 8 | - |
| **ICS Only (%)** | 7 | 0 | - |
| **ICS + LABA (%)** | 7 | 8 | - |
| **ICS + LAMA (%)** | 0 | 0 | - |
| **LABA + LAMA (%)** | 0 | 0 | - |
| **Triple (%)** | 67 | 80 | - |
| **No inhaled medication (%)** | 13 | 0 | - |
| **CRP** | 3 [1-28] | 3 [1-157] | 0.65 |

Patients were categorised into two groups based on bacterial load defined by genome copies/ml of ≥1x10^4^ at baseline and 6 months later; NO-NO (No colonisation at either visit) and HI-HI (Presence of *Haemophilus influenzae* at both visits, including those with ≥1 PPM). Statistical analysis was performed using Chi-squared, students t-test and Mann-Whitney U as appropriate. Summaries are presented as percentages, Mean (SD) and Median [Range] as appropriate (n = 55 ^‡^).

* = significant difference (*p<*0.05)

^‡^ The following data were not available for; n=3 exacerbation data (between visits), n=12 exacerbation data (1 year period) at 12 months, and n=8 CRP

**Table S17: Baseline sputum inflammatory cell counts for different PPM groups, including those with *H.influenzae* + ≥1 PPM.**

|  | **NO-NO** | **HI-HI** | ***P value*** |
| --- | --- | --- | --- |
| **Characteristic** | n=30 | n=25 |  |
| **Sputum total cell count x10^6^/g** | 3.01 [0.31-22.15] | 7.14 [0.54-84.56] | 0.12 |
| **Sputum Neutrophil (%)** | 68.13 [1-96.25] | ****83.25 [48.38-99.25]** | **<0.01** |
| **Sputum Eosinophil (%)** | 2.13 [0-17] | ***0.50 [0.00-3.50]** | **0.03** |
| **Sputum Lymphocyte (%)** | 0.05 [0-6] | 0.00 [0.00-1.00] | 0.15 |
| **Sputum Macrophage (%)** | 18.67 [2-85.5] | ****8.82 [0.50-46.88]** | **<0.01** |
| **Sputum Epithelial Cells (%)** | 4.18 [0-31] | 1.0 [0.00-18.00] | 0.22 |
| **Sputum Neutrophil cell count x10^6^/g** | 1.87 [0-19.88] | 5.90 [0.37-82.44] | 0.08 |
| **Sputum Eosinophil cell count x10^6^/g** | 0.08 [0-0.66] | 0.04 [0.00-0.42] | 0.33 |
| **Sputum Lymphocyte cell count x10^6^/g** | 0 [0-0.18] | 0.00 [0.00-0.09] | 0.64 |
| **Sputum Macrophage cell count x10^6^/g** | 0.51 [0.14-2.16] | 0.311 [0.08-4.42] | 0.42 |
| **Sputum Epithelial cell count x10^6^/g** | 0.1 [0-0.41] | 0.13 [0.00-1.30] | 0.48 |
| **Sputum Eosinophil ≥3% (%)** | 40 | ****8** | **<0.01** |
| **Sputum Eosinophil ≥2% (%)** | 60 | ****20** | **0.01** |

Patients were categorised into two groups based on bacterial load defined by genome copies/ml of ≥1x10^4^ at baseline and 6 months later; NO-NO (No colonisation at either visit) and HI-HI (Presence of *Haemophilus influenzae* at both visits, including those with ≥1 PPM). Statistical analysis was performed using Chi-squared, students t-test and Mann-Whitney U as appropriate. Summaries are presented as percentages, Mean (SD) and Median [Range] as appropriate (n = 55^‡^).

*, ** = significant difference (*p<*0.05 and <0.01 respectively)

^‡^ The following data were not available; 19 patients had no absolute sputum cell counts available 12 patients had no sputum cell count % available.

**Table S18: Occurrence and average relative abundance of contaminate genera detected in sequenced negative ‘blank’ controls by Salter et al (**[**5**](#_ENREF_5)**) in the COPDMAP dataset.**

|  | **Occurrence**  **(relative abundance > 0)** | **Occurrence**  **(relative abundance > 0.1)** | **Average relative abundance** |
| --- | --- | --- | --- |
| ***Alphaproteobacteria*** |  |  |  |
| *Afipia* | 0 | 0 | 0 |
| *Aquabacterium* | 0 | 0 | 0 |
| *Asticcacaulis* | 0.002793296 | 0 | 6.06E-08 |
| *Aurantimonas* | 0.025139665 | 0 | 6.97E-07 |
| *Beijerinckia* | 0 | 0 | 0 |
| *Bosea* | 0.001396648 | 0 | 6.06E-08 |
| *Bradyhizobium* | 0 | 0 | 0 |
| *Brevundimonas* | 0.044692737 | 0 | 1.97E-05 |
| *Caulobacter* | 0.001396648 | 0 | 3.03E-08 |
| *Craurococcus* | 0 | 0 | 0 |
| *Devosia* | 0.011173184 | 0 | 4.25E-07 |
| *Hoeflea* | 0 | 0 | 0 |
| *Mesorhizobium* | 0 | 0 | 0 |
| *Methylobacterium* | 0.118715084 | 0 | 4.52E-06 |
| *Novosphingobioum* | 0 | 0 | 0 |
| *Ochrobactrum* | 0.698324022 | 0 | 4.58E-05 |
| *Paracoccus* | 0.086592179 | 0 | 2.30E-06 |
| *Pedomicrobiom* | 0 | 0 | 0 |
| *Phyllobacterium* | 0.009776536 | 0 | 3.34E-07 |
| *Rhizobium* | 0.005586592 | 0 | 1.21E-07 |
| *Roseomonas* | 0 | 0 | 0 |
| *Sphingobium* | 0.036312849 | 0 | 6.22E-06 |
| *Sphingomonas* | 0.160614525 | 0 | 3.02E-05 |
| *Sphingopyxis* | 0.018156425 | 0 | 8.49E-07 |
| ***Betaproteobacteria*** |  |  |  |
| *Acidovorax* | 0.019553073 | 0 | 7.28E-07 |
| *Azoarcus* | 0 | 0 | 0 |
| *Azospira* | 0 | 0 | 0 |
| *Burkholderia* | 0 | 0 | 0 |
| *Comamonas* | 0.008379888 | 0 | 1.82E-07 |
| *Cupriavidus* | 0.001396648 | 0 | 9.10E-08 |
| *Curvibacter* | 0 | 0 | 0 |
| *Delftia* | 0.005586592 | 0 | 1.52E-07 |
| *Duganella* | 0 | 0 | 0 |
| *Herbaspirillum* | 0 | 0 | 0 |
| *Janthinobacterium* | 0.002793296 | 0 | 1.52E-07 |
| *Kingella* | 0.995810056 | 0 | 0.000529353 |
| *Leptothrix* | 0 | 0 | 0 |
| *Limnobacter* | 0 | 0 | 0 |
| *Massilia* | 0 | 0 | 0 |
| *Methylophilus* | 0 | 0 | 0 |
| *Methyloversatilis* | 0 | 0 | 0 |
| *Oxalobacter* | 0.025139665 | 0 | 1.27E-06 |
| *Pelomonas* | 0 | 0 | 0 |
| *Polaromonas* | 0 | 0 | 0 |
| *Ralstonia* | 0.005586592 | 0 | 1.21E-07 |
| *Schlegelella* | 0 | 0 | 0 |
| *Sulfuritalea* | 0 | 0 | 0 |
| *Undibacterium* | 0 | 0 | 0 |
| *Variovorax* | 0.906424581 | 0 | 0.000159964 |
| ***Gammaproteobacteria*** |  |  |  |
| *Acinetobacter* | 1 | 0 | 0.001896829 |
| *Enhydrobacter* | 0.083798883 | 0 | 3.49E-06 |
| *Enterobacter* | 0.997206704 | 0 | 0.000509339 |
| *Escherichia* | 0.048882682 | 0 | 1.09E-06 |
| *Nevskia* | 0.001396648 | 0 | 6.06E-08 |
| *Pseudomonas* | 0.995810056 | 0.004189944 | 0.004687729 |
| *Pseudoxanthomonas* | 0.019553073 | 0 | 8.79E-07 |
| *Psychobacter* | 0 | 0 | 0 |
| *Stenotrophomonas* | 0.780726257 | 0 | 0.000139556 |
| *Xanthomonas* | 0 | 0 | 0 |
| ***Actinobacteria*** |  |  |  |
| *Aeromicrobium* | 0 | 0 | 0 |
| *Arthrobacter* | 0.005586592 | 0 | 2.73E-07 |
| *Beutenbergia* | 0 | 0 | 0 |
| *Brevibacterium* | 0.027932961 | 0 | 1.27E-06 |
| *Corynebacterium* | 1 | 0 | 0.001012521 |
| *Curtobacterium* | 0 | 0 | 0 |
| *Dietzia* | 0.002793296 | 0 | 2.12E-07 |
| *Geodermatophilus* | 0 | 0 | 0 |
| *Janibacter* | 0.018156425 | 0 | 4.25E-07 |
| *Kocuria* | 0 | 0 | 0 |
| *Microbacterium* | 0.159217877 | 0 | 6.22E-06 |
| *Micrococcus* | 0.054469274 | 0 | 2.03E-06 |
| *Microlunatus* | 0.002793296 | 0 | 9.10E-08 |
| *Patulibacter* | 0 | 0 | 0 |
| *Propionibacterum* | 0 | 0 | 0 |
| *Rhodococcus* | 0.909217877 | 0 | 0.000154081 |
| *Tsukamurella* | 0 | 0 | 0 |
| ***Firmicutes*** |  |  |  |
| *Abiotrophia* | 0 | 0 | 0 |
| *Bacillus* | 0.048882682 | 0 | 2.03E-06 |
| *Brevibacillus* | 0 | 0 | 0 |
| *Brochothrix* | 0.002793296 | 0 | 9.10E-08 |
| *Facklamia* | 0.002793296 | 0 | 9.10E-08 |
| *Paenibacillus* | 0.060055866 | 0 | 2.30E-06 |
| *Streptococcus* | 1 | 0.032122905 | 0.044335024 |
| ***Bacteroidetes*** |  |  |  |
| *Chryseobacterium* | 0.205307263 | 0 | 1.11E-05 |
| *Dyadobacter* | 0.002793296 | 0 | 6.06E-08 |
| *Flavobacterium* | 0.026536313 | 0 | 1.46E-06 |
| *Hydrotalea* | 0 | 0 | 0 |
| *Niatella* | 0 | 0 | 0 |
| *Olivibacter* | 0 | 0 | 0 |
| *Pedobacter* | 0.009776536 | 0 | 4.25E-07 |
| *Wautersiella* | 0 | 0 | 0 |
| ***Deinococcus-Thermus*** | | | |
| *Deinococcus* | 0.124301676 | 0 | 5.34E-06 |

The first column (occurrence relative abundance > 0) was calculated as the fraction of samples in which each genus has abundance greater than 0. The second column (occurrence relative abundance > 0.1) was calculated as the fraction of samples in which each genus has abundance greater than 0.1. And the third column is the average relative abundance of each genus across all samples.
